# Copper(ii) curcumin complexes for endoplasmic reticulum targeted photocytotoxicity†

**DOI:** 10.1039/d2ra04813b

**Published:** 2022-10-27

**Authors:** Atrayee Banaspati, Vanitha Ramu, Md Kausar Raza, Tridib K. Goswami

**Affiliations:** Department of Chemistry, Gauhati University Guwahati 781014 Assam India tridibgoswami05@gmail.com; Department of Inorganic and Physical Chemistry, Indian Institute of Science Bangalore 560012 India kausarraza91@gmail.com

## Abstract

Three copper(ii) complexes *viz.* [Cu(cur)(L)(ClO_4_)] (1–3), where Hcur is curcumin and L is 1,10-phenanthroline (phen, 1), dipyrido[3,2-*d*:2′,3′-*f*]quinoxaline (dpq, 2), or dipyrido[3,2-*a*:2′,3′-*c*]phenazine (dppz, 3) were synthesized, fully characterized by various physicochemical methods and evaluated for their light-assisted chemotherapeutic potential. The complexes [Cu(acac)(L)(ClO_4_)] (4–6), where Hacac is acetylacetone and L is phen (in 4), dpq (in 5) and dppz (in 6), were synthesized and used as controls. The solid state structures of complexes 4 and 5 were determined by single crystal X-ray diffraction. The curcumin complexes (1–3) were redox inactive at the copper centre, whereas the acetylacetonato complexes (4–6) displayed a Cu(ii)/Cu(i) couple at ∼0.1 V *vs.* Ag/AgCl reference electrode in DMF. Complexes 1–3 showed an intense curcumin-based band at ∼440 nm in DMF–Tris–HCl buffer (pH = 7.2) (1 : 9 v/v) which masks the copper based d–d band. The complexes bind to human serum albumin (HSA) with moderate efficacy. They also displayed significant binding affinity for calf-thymus (CT) DNA. The lipophilic curcumin complexes show remarkable visible light induced cytotoxicity (IC_50_ = ∼4 μM) with high phototoxic indices (PI) with low dark toxicity in human cervical carcinoma (HeLa) and human lung carcinoma (A549) cells. The corresponding acetylacetonato controls (4–6) did not show significant cytotoxicity in the dark or light. DCFDA and annexin V-FITC/PI assays using flow cytometry confirm the induction of significant apoptosis in cancer cells *via* generation of cytotoxic reactive oxygen species upon photoactivation. Confocal microscopic images using complex 3 demonstrate localization of the complexes predominantly in the endoplasmic reticulum of HeLa cells.

## Abstract 2

Cu(ii) curcumin complexes show significant photo-enhanced cytotoxicity in HeLa and A549 cancer cells in visible light *via* an apoptosis mechanism through generation of ROS with low dark toxicity.

## Graphical abstract

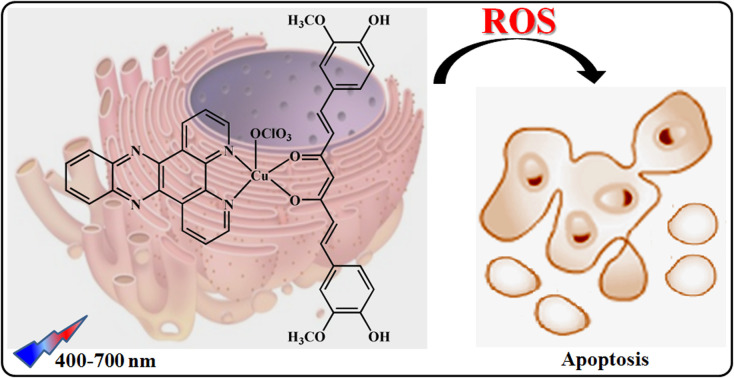

## 1. Introduction

Curcumin [(1*E*,6*E*)-1,7-bis(4-hydroxy-3-methoxyphenyl)-1,6-heptadiene-3,5-dione], a naturally occurring polyphenol that is major constituent of traditional Indian and Chinese medicine and spice, has been extensively investigated for the treatments of various ailments such as antitumoral, anti-inflammatory, antimicrobial, antioxidant, antihepatotoxic, antihyperlipidemic, antiviral, anti-Alzheimer's disease, *etc.*^1–5^ This highly pleiotropic molecule is known to show its anticancer activity by inhibiting various vital enzymatic pathways.^3–9^ However, poor bioavailability as a result of instability in biological fluid has remained a major obstacle for realising the medicinal use of curcumin despite having enormous potential for the prevention and cure of various diseases.^10–12^ Curcumin has been recently labelled as a PAINS (pan assay interference compounds) and IMPS (invalid metabolic panaceas) compound because of its poor pharmacokinetic and pharmacodynamic profiles.^13^ Scientists around the globe are trying to address the issues of instability and bioavailability by various methods such as controlled release of the molecule from various materials, covalent organic modifications, chelation to metals, *etc.* Amongst these, chelation to metal is particularly attractive as the central β-diketo moiety of the natural polyphenol is a very effective chelator to various metals to form coordination complexes, which can potentially reduce the problem of instability to a great extent.^4,10^

Research in the field of metal based anticancer agents started with the success of cisplatin and its next generation drugs in clinics.^14,15^ Unfortunately, high intrinsic toxicity and resistant developed by tumours limits their use in clinic and are only used in combination therapy with other organic anticancer drugs.^16–18^ This has prompted design, synthesis and evaluation antitumor potential of various bio-essential first row transition metals that are expected to have lesser toxicity compared to that of cisplatin.^19–22^ However, not many attempts have shown promise in this direction so far. An alternative strategy to reduce the side effects of metal-based anticancer drugs is to photoactivate them to generate cytotoxic species, which are otherwise non-toxic in the dark.^23,24^ This strategy was inspired by the success of the Photofrin®, a hematoporphyrin derivative of variable composition with limited clinical applications for the treatment of selected type of cancers.^25–27^ Curcumin molecule have excellent photophysical properties.^28^ It absorbs in the range ∼408–430 nm and emits in green (∼460–560 nm) depending on the nature of the solvent.^4,6^ This prompted us to explore the potential of metal-chelated curcumin in the design of inorganic photodynamic therapy agents.

**Chart 1:**
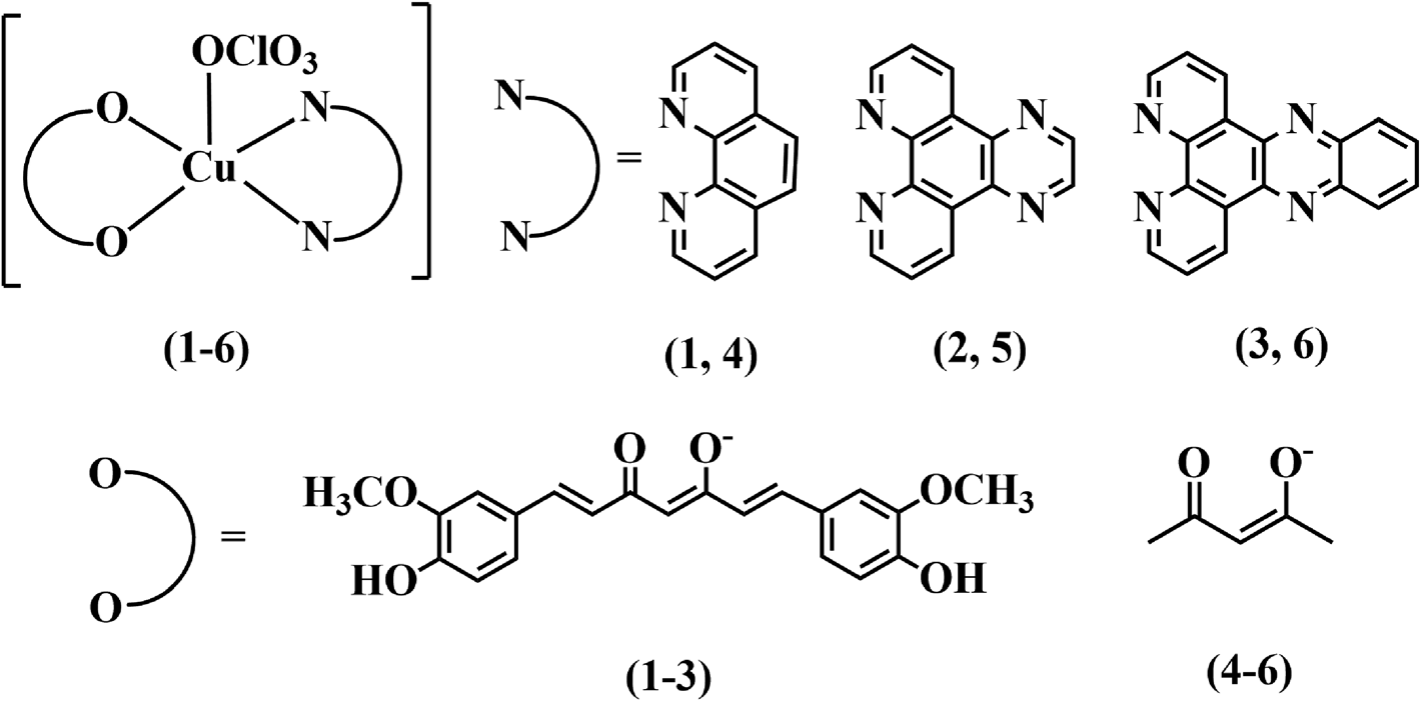
Schematic drawings of the complexes [Cu(Cur)(L)(ClO_4_)] (1–3) and [Cu(acac)(L)(ClO_4_)] (4–6) (L = phen, 1, 4; dpq, 2, 5; dppz, 3, 6).

In this work, we report the design and synthesis of three simple ternary Cu(ii) complexes having curcumin along with phenanthroline bases as ligands, *viz.* [Cu(Cur)(L)(ClO_4_)] (1–3), where HCur is the curcumin and L is 1,10-phenanthroline (phen in 1), dipyrido[3,2-*d*:2′,3′-*f*]quinoxaline (dpq in 2), dipyrido[3,2-*a*:2′,3′-*c*]phenazine (dppz in 3) (Chart 1) and their photocytotoxicity in cancer cells. Three control complexes, namely[Cu(acac)(L)(ClO_4_)] (4–6) having acetylacetonato ligand (acac) along with phenanthroline bases (phen in 4; dpq in 5 and dppz in 6) in their formulations, were prepared to evaluate the efficacies of the curcumin complexes in killing cancer cells under irradiation of visible light. The toxicity of the complexes against human cervical carcinoma (HeLa) and human lung carcinoma (A549) cancer cell lines were tested in both dark as well as in visible light. The complexes having curcumin were found to be highly photo-cytotoxic (IC_50_ values) in HeLa and A549 cells, with minimal dark cytotoxicity. DCFDA assay shows the generation of ROS as the cytotoxic species. Annexin-V/FITC/PI assay suggests apoptosis as the predominant cell death pathway induced by the complexes under visible light illumination. The fluorescence property of curcumin is used to track these potential inorganic PDT anticancer agents inside the cells. Confocal microscopic images display that the curcumin complexes selectively localize in the endoplasmic reticulum of HeLa cells.

## 2. Experimental section

### 2.1 Materials and measurements

All the reagents and chemicals were obtained from commercial sources (SD Fine Chemicals, India; LobaChemie, India; Sigma-Aldrich, U.S.A.) and used as such. Tris-(hydroxymethyl)aminomethane–HCl (Tris–HCl) buffer solution was prepared using deionized and sonicated double distilled water. Calf thymus DNA, human serum albumin (HSA), Dulbecco's Modified Eagle's Medium (DMEM), MTT (3-(4,5-dimethylthiazol-2-yl)-2,5-diphenyltetrazolium bromide), 2′,7′-dichlorofluorescein diacetate (DCFDA), 4′,6-diamidino-2-phenylindole (DAPI), ER-tracker red propidium iodide (PI), Dulbecco's phosphate buffered saline (DPBS) and fetal bovine serum (FBS) were purchased from Sigma (U.S.A.). Curcumin was purchased from LobaChemie (India). Dipyrido[3,2-*d*:2′,3′-*f*]quinoxaline (dpq) and dipyrido[3,2-*a*:2′,3′-*c*]phenazine (dppz) were prepared by reported literature procedures using 1,10-phenanthroline-5,6-dione as a precursor reacted with ethylenediamine and 1,2-phenylenediamine, respectively.^29,30^

The elemental analysis was carried out using a ThermoFinnigan Flash EA 1112 CHN analyzer. The infrared, UV-visible and emission spectra were recorded on Shimadzu IRAffinity-1S, Shimadzu UV-1800 and Hitachi F-2500 spectrophotometer, respectively. Room-temperature magnetic susceptibility data were obtained from a Sherwood Scientific, Cambridge, England, using Hg[Co(NCS)_4_] as a standard. Experimental susceptibility data were corrected for diamagnetic contributions.^31^ Cyclic voltammetric measurements were carried out at 25 °C on a CHI660D (CH Instruments, Inc., USA) workstation using a three electrode set-up with a glassy carbon working, platinum wire auxiliary and an Ag/AgCl reference electrode. Tetrabutylammonium perchlorate (TBAP) (0.1 M) was used as a supporting electrolyte in DMF. The electrochemical data were uncorrected for junction potentials. Electrospray ionization mass spectral measurements were done using Agilent 6538 Ultra High Definition Accurate Mass-Q-TOF (LC-HRMS) and Bruker Daltonics make Esquire 300 Plus ESI model mass spectrometers. All experiments requiring light exposure were performed with a broad-band white light using Luzchem Photoreactor (model LZC-1, Ontario, Canada) fitted with eight fluorescent Sylvania white tubes (*λ* = 400–700 nm) and standard protocols. Cytotoxicity data were obtained with a TECAN microplate reader and fitted using GraphPad Prism 7 software. Flow cytometric experiments were performed using fluorescence-activated cell sorting (FACS) Verse instrument (BD Biosciences) fitted with a MoFLo XDP cell sorter and analyzer with three lasers (*λ* = 488, 365, and 640 nm) and 10-color parameters. Confocal microscopy images were acquired from Leica laser scanning confocal microscope (TCS, SP5 DM6000) with an oil immersion lens with magnification of 63×. Images were processed by using LAS AF Lite software.

### 2.2 Synthesis

#### 2.2.1 Synthesis of [Cu(Cur)(L)(ClO_4_)] (L = phen, 1; dpq, 2; dppz, 3)

Complexes 1–3 were prepared by a general method in which copper(ii)acetate monohydrate (0.1 g, 0.5 mmol) was dissolved in 5 mL of methanol. To this solution was added a methanolic solution of the heterocyclic base (L: phen, 0.1 g; dpq, 0.12 g; dppz, 0.14 g, 0.5 mmol) and stirred at room temperature for 0.5 h. This was followed by addition of a methanolic solution of curcumin (Hcur 0.18, 0.5 mmol) and stirred for another 1 h. The complexes, precipitated as brick-red solids as their perchlorate salts upon addition of a saturated solution of NaClO_4_ in methanol, were thoroughly washed with water, cold methanol and finally dried *in vacuo* over anhydrous CaCl_2_ (yield: 0.28 g 79% for 1; 0.32 g 84% for 2; 0.36 g 88% for 3) (Scheme S1, ESI†).

Anal. calcd for C_33_H_27_N_2_O_10_CuCl (1): C, 55.78; H, 3.83; N, 3.94. Found: C, 55.61; H, 4.02; N, 3.99. Selected IR data (KBr phase, cm^−1^): 3416br, 2973m, 2930w, 1621m, 1560s (C=O), 1507m (C=C), 1464m, 1423m, 1292m, 1177m, 1058s (ClO_4_^−^), 970m, 813w, 720m, 620m. ESI-MS in MeOH: *m*/*z* = 610.1166 [M–(ClO_4_^−^)]^+^. UV-visible in DMF [*λ*_max_/nm (*ε*/M^−1^ cm^−1^)]: 438 (15 200), 271 (17 200). *μ*_eff_ = 1.81*μ*_B_ at 298 K.

Anal. calcd for C_35_H_27_N_4_O_10_CuCl (2): C, 55.12; H, 3.57; N, 7.35. Found: C, 54.93; H, 3.64; N, 7.25. Selected IR data (KBr phase, cm^−1^): 3434br, 1952w, 1722w, 1627m, 1590m (C=O), 1516s (C=C), 1393m, 1280m, 1118s, 1080s (ClO_4_^−^), 960w, 815w, 726m, 631s. ESI-MS in MeOH: *m*/*z* = 662.1243 [M–(ClO_4_^−^)]^+^. UV-visible in DMF [*λ*_max_/nm (*ε*/M^−1^ cm^−1^)]: 448 (28 400), 297 (29 600), 257 (46 400). *μ*_eff_ = 1.79*μ*_B_ at 298 K.

Anal. calcd for C_39_H_29_N_4_O_10_CuCl (3): C, 57.64; H, 3.60; N, 6.89. Found: C, 57.39; H, 3.48; N, 6.81. Selected IR data (KBr phase, cm^−1^): 3412br, 2969w, 2290w, 1619m, 1586m (C=O), 1511vs (C=C), 1391m, 1289vs, 1206w, 1157m, 1073s (ClO_4_^−^), 964w, 815m, 726m, 616m. ESI-MS in MeOH: *m*/*z* = 712.1377 [M–(ClO_4_^−^)]^+^. UV-visible in DMF [*λ*_max_/nm (*ε*/M^−1^ cm^−1^)]: 438 (14 800), 384sh (11 600), 283 (27 600). *μ*_eff_ = 1.80*μ*_B_ at 298 K.

#### 2.2.2 Synthesis of [Cu(acac)(L)(ClO_4_)] (L = phen, 1; dpq, 2; dppz, 3)

The complexes 4–6 were prepared in a similar way as described for 1–3 (0.5 mmol scale) except that acetylacetone (Hacac 50 μL, 0.5 mmol) was used instead of Hcur. The complexes were isolated as blue solids as their perchlorate salts (yield: 0.19 g, 86% for 4; 0.22 g, 89% for 5; 0.24 g, 88% for 6) (Scheme S2, ESI†).

Anal. calcd for C_17_H_15_N_2_O_6_CuCl (4): C, 46.16; H, 3.42; N, 6.33. Found: C, 46.05; H, 3.49; N, 6.22. Selected IR data (KBr phase, cm^−1^): 3405br, 2988w, 1585vs (C=O), 1514vs (C=C), 1421s, 1381s, 1273m, 1223m, 1145s, 1081vs (ClO_4_^−^), 1017m, 925m, 854s, 770m, 718s, 626s. ESI-MS in MeOH: *m*/*z* = 342.0459 [M–(ClO_4_^−^)]^+^. UV-visible in DMF [*λ*_max_/nm (*ε*/M^−1^ cm^−1^)]: 616 (30), 443 (70), 271 (26 800). *μ*_eff_ = 1.78*μ*_B_ at 298 K.

Anal. calcd for C_19_H_15_N_4_O_6_CuCl (5): C, 46.16; H, 3.06; N, 11.33, Found: C, 45.98; H, 3.27; N, 11.16. Selected IR data (KBr phase, cm^−1^): 3416br, 2975w, 2930w, 1566vs (C=O), 1514vs (C=C), 1478s, 1378vs, 1278m, 1214m, 1097vs (ClO_4_^−^), 1056s, 927m, 877w, 820s, 727s, 620s. ESI-MS in MeOH: *m*/*z* = 394.0498 [M–(ClO_4_^−^)]^+^. UV-visible in DMF [*λ*_max_/nm (*ε*/M^−1^ cm^−1^)]: 621 (60), 336sh (3800), 293 (18 400), 257 (36 000). *μ*_eff_ = 1.79*μ*_B_ at 298 K.

Anal. calcd for C_23_H_17_N_4_O_6_CuCl (6): C, 50.74; H, 3.15; N, 10.29. Found: C, 50.68; H, 3.07; N, 10.51. Selected IR data (KBr phase, cm^−1^): 3416br, 2975w, 1571vs (C=O), 1520vs (C=C), 1464w, 1420s, 1385s, 1277m, 1235m, 1191m, 1077s (ClO_4_^−^), 920m, 759s, 770s, 727vs, 620vs. ESI-MS in MeOH: *m*/*z* = 444.0656 [M–(ClO_4_^−^)]^+^. UV-visible in DMF [*λ*_max_/nm (*ε*/M^−1^ cm^−1^)]: 606 (40), 437 (100), 377 (6400), 360 (6400), 275 (30 800). *μ*_eff_ = 1.79*μ*_B_ at 298 K.

### 2.3 X-ray crystallographic procedure

The crystal structures of [Cu(Cur)(phen)(ClO_4_)] (4) and [Cu(Cur)(dpq)(ClO_4_)] (5) were obtained by single crystal X-ray diffraction method. Crystals of 4 and 5 were obtained from the methanolic solution of the complex on slow evaporation of the solvent for 2–3 days. Crystal mounting was done on glass fibres with epoxy cement. All geometric and intensity data were collected at room temperature using an automated Bruker SMART APEX CCD diffractometer equipped with a fine focus 1.75 kW sealed tube Mo-K_α_ X-ray source (*λ* = 0.71073 Å) with increasing *ω* (width of 0.3° per frame) at a scan speed of 5 s per frame. Intensity data, collected using an *ω*–2*θ* scan mode, were corrected for Lorentz-polarization effects and for absorption.^32^ The structure solution was done by the combination of Patterson and Fourier techniques and refined by full-matrix least-squares method using SHELX system of programs.^33^ All hydrogen atoms belonging to the complex were in their calculated positions and refined using a riding model. All non-hydrogen atoms were refined anisotropically. The perspective views of the molecules were obtained by ORTEP.^34^

### 2.4 DNA and protein binding experiments

The DNA binding experiments were performed using calf thymus (CT) DNA by following reported procedures.^35^ The complex solution (25 μM) in 5 mM Tris–HCl buffer (pH 7.2) containing 20% DMF was titrated with the 220 μM DNA and the intensity of the band at ∼260–280 nm was monitored for the complexes. The spectra were recorded after equilibration for 5 min allowing the complexes to bind to the DNA. The intrinsic equilibrium binding constant (*K*_b_) and the fitting parameter (*s*) of the complexes to DNA were obtained by McGhee–von Hippel (MvH) method using the expression of Bard and coworkers.^36,37^

The protein binding study was carried out by tryptophan fluorescence quenching experiments using human serum albumin (HSA, 2 μM) in Tris–HCl buffer (pH 7.2). Quenching of the emission intensity of tryptophan residue (Trp 214) of HSA at 320 nm (excitation wavelength at 280 nm) was monitored using complexes 1–6 as quenchers with increasing concentration.^38^*I*_0_/*I vs.* [complex] plots were constructed. Linear fit of the data using the equation *I*_0_/*I* = 1 + *K*[Q], where *I*_0_ and *I* are the emission intensity of HSA in the absence of quencher and emission intensity of HSA in the presence of the quencher of concentration [Q] gave the binding constant *K*_HSA_ values using OriginPro 8. The static or dynamic nature of fluorescence quenching of HSA exhibited by complex 3 was analyzed by fluorescence lifetime measurement experiments. Average fluorescence lifetime of pure HSA (2 μM) was measured and compared with its fluorescence lifetime in presence of two different concentrations (10 and 20 μM) of complex 3 using a time-resolved fluorimeter.^39^

### 2.5 Cell culture

The human cancer cell lines, HeLa (human cervical carcinoma) and A549 (human lung carcinoma), available in-house at the Indian Institute of Science, Bangalore, India were used for the experiments. The cells were maintained in Dulbecco's Modified Eagle's Medium (DMEM), supplemented with 10% fetal bovine serum (FBS), 100 IU mL^−1^ of penicillin, 100 mg mL^−1^ of streptomycin and 2 mM Glutamax at 37 °C in a humidified incubator at 5% CO_2_. The adherent cultures were grown as monolayer and were passaged once in 4–5 days by trypsinizing with 0.25% Trypsin–EDTA.

### 2.6 Cell viability assay

The photocytotoxicity of the complexes was assessed using MTT assay based on the ability of mitochondrial dehydrogenases in the viable cells to cleave the tetrazolium rings of MTT and forming dark blue membrane impermeable crystals of formazan that were measured at 570 nm giving an estimate of the number of viable cells.^40^ Approximately, 1 × 10^4^ cells of HeLa and A549 were plated in a 96-well culture plate in DMEM supplemented with 10% fetal bovine serum and cultured overnight. Different concentrations of the complexes were added to the cells, and incubation was continued for 4 h in dark. After incubation, the medium was replaced with 50 mM phosphate buffer, pH 7.4, containing 150 mM NaCl (PBS) and photo-irradiation was done for 1 h in visible light of 400–700 nm using Luzchem Photoreactor (Model LZC-1, Ontario, Canada; light fluence rate: 2.4 mW cm^−2^; light dose = 10 J cm^−2^). PBS was replaced with 10% DMEM after irradiation. Incubation was continued for a further period of 19 h in dark for light exposed cells and for 20 h in dark for light unexposed cells followed by addition of 25 μL of 4 mg mL^−1^ of MTT to each well and incubated for an additional 3 h. The culture medium was discarded, and a 100 μL volume of DMSO was added to dissolve the formazan crystals. The absorbance at 570 nm was determined using an ELISA microplate reader (BioRad, Hercules, CA, USA). The cytotoxicity of the test compounds was measured as the percentage ratio of the absorbance of the treated cells over the untreated controls. The IC_50_ values were determined by nonlinear regression analysis (Graphpad Prism, version 7).

### 2.7 Partition coefficient of the complexes between *n*-octanol/water and cellular uptake by flow-cytometry

A modified shake-flask method was used to determine the partition coefficients of the complexes 1–6 between *n*-octanol and water.^41^ Briefly, 5 ml solution of a complex in octanol-saturated water was shaken with 5 ml of water-saturated *n*-octanol in a falcon tube for ∼30 min using a vortex. The biphasic mixture was centrifuged for ∼5 min at 4000 rpm and the layers were separated. The concentration of the complexes 1–6 in each layer was measured by UV-visible spectroscopy. The partition coefficients of 1–6 were determined at three different concentrations and averaged out.

Uptake of the complexes 1–3 in HeLa cells were studied by flow cytometric method reported in literature.^42^ HeLa cells were treated with 1–3 (10 μM) and incubated for 4 h in dark. The cells were harvested by trypsinization and a suspension in PBS was prepared. The uptake of the green fluorescent complexes in HeLa cells were evaluated by flow cytometry.

### 2.8 DCFDA assay for detection of ROS

The generation of cellular reactive oxygen species (ROS) was assessed using DCFDA assay.^43^ Approximately ∼1 × 10^6^ HeLa cells were incubated with complex 3 (5 μM) for 4 h which is followed by photo-irradiation (400–700 nm) for 1 h in PBS. The cells were then harvested by trypsinization and a single cell suspension in PBS was made. After that the cells were treated with DCFDA solution (10 μM, in DMSO) in dark at room temperature for 5 min. The distribution of DCFDA stained HeLa cells was determined by flow cytometry in the FL-1 channel.

### 2.9 Annexin-V/FITC/PI assay

The mechanism of light-assisted cell death induced by the complexes was studied by annexin-V/FITC/PI assay using complexes 1 and 3 in 1% DMSO/DMEM.^44^ ∼4 × 10^5^ HeLa cells seeded in six-well plates were cultured for 24 h. The cells were then incubated with 3 (5 μM) for 4 h in the dark and then exposed to visible light (*λ* = 400–700 nm, light dose = 10 J cm^−2^) in phenol red free media for 1 h. The corresponding dark control experiment was carried out. Cells were incubated for another 19 h and 20 h respectively for the light exposed and unexposed plates in DMEM/10% fetal bovine serum (FBS) buffer in the dark. The medium was discarded, cells were trypsinized and re-suspended in 140 μL Annexin V binding buffer (100 mM HEPES/NaOH, pH 7.4 containing 140 mM NaCl and 2.5 mM CaCl_2_). Annexin-V/FITC (0.5 mL) and propidium iodide (PI; 1 mL) were added to the cell suspensions, incubated for 5 min and readings were taken using a flow cytometer.

### 2.10 Confocal microscopy

For the confocal laser scanning microscopy, approximately 1 × 10^5^ HeLa cells were seeded on 35 mm round glass bottom dishes. After 24 h, the cells were incubated with compound 3 (10 μM) in 1% DMSO/DMEM for 4 h. The cells were washed with DPBS and treated with ER-tracker red (500 nM, 30 min) and 4′,6-diamidino-2-phenylindole (DAPI) as endoplasmic reticulum and nucleus selective trackers respectively in serum-free medium for 30 min at 37 °C. The images were recorded in fresh DMEM in the dark by using Zeiss LSM 880 with Airyscan with an oil immersion lens having magnification 63×. For the incubated cells with compound 3, the CLSM images were captured with a band path of 650–750 nm on excitation at 633 nm. The fluorescence of ER-tracker red in HeLa cells were captured with a band path of 520–560 nm upon excitation at 508 nm and for 4′,6-diamidino-2-phenylindole (DAPI), the emission detection band path was set to 420–460 nm with excitation at 408 nm. The images were analyzed with the help of LAS AF Lite software.^45^

## 3. Results and discussion

### 3.1 Synthesis and general aspects

**Fig. 1:**
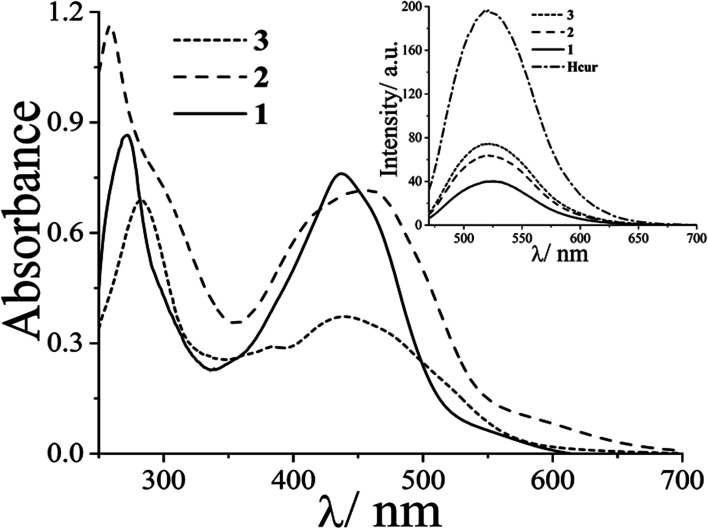
The electronic spectra of [Cu(Cur)(phen)(ClO_4_)] (1, —); [Cu(Cur)(dpq)(ClO_4_)] (2, ----); and [Cu(Cur)(dppz)(ClO_4_)] (3, ····) (25 μM) in DMF–Tris–HCl buffer (pH 7.2) (1 : 4 v/v). The inset shows the emission spectra of the complexes 1–3 (5 μM) and curcumin (Hcur) in the same solvent mixture.

Ternary copper(ii) complexes [Cu(Cur)(L)(ClO_4_)] (1–3) having curcumin (HCur) in mono-anionic form and *N*,*N*-donor phenanthroline bases (L: phen, 1; dpq, 2; dppz, 3) were synthesized in good yield (∼80%) from the reaction of copper(ii) acetate monohydrate and the respective phenanthroline bases followed by addition of curcumin in methanol (Scheme S1, ESI†). The complexes [Cu(acac)(L)(ClO_4_)] (1–3) (L: phen, 4; dpq, 5; dppz, 6) were prepared using similar procedures and were used as controls. Coordination to copper not only enhances the stability of curcumin at physiological pH, but also make the compounds lipophilic to pass through the lipid bilayer of cells. The phenanthroline bases incorporated in the structures make them more lipophilic and capable of binding to vital biomolecules such as DNA and serum protein.^46^ Whereas, DNA could be a potential target of these molecules, human serum albumin (HSA) a potential career. The complexes were characterized by various spectroscopic and analytical techniques. Selected physicochemical data are given in Table 1. The solution phase stability of the complexes 1–6 was also evident from their ESI-MS spectra showing essentially the base peak as [M–(ClO_4_^−^)]^+^ in methanol (Fig. S1–S6, ESI†). The theoretical and experimental isotopic distributions of the base peaks were found to be in good agreement. In IR spectra, characteristic bands due to C=O and C=C stretching vibrations of curcumin and acetylacetonato ligand were observed at ∼1590 and ∼1500 cm^−1^ respectively (Fig. S7–S12, ESI†). In addition, all the complexes displayed a band ∼1100 cm^−1^ due to ClO_4_^−^ stretching. The signal due to ClO_4_^−^ split due to its coordination to the metal center.^47^ The presence of one-electron paramagnetic 3d^9^-Cu(ii) was confirmed by the magnetic moment values of ∼1.8 *μ*_B_ at 25 °C. The UV-visible spectra of 1–3 in DMF–Tris–HCl buffer (1 : 4 v/v), showed an intense band at ∼440 nm due to π → π* transition in curcumin (Fig. 1). This band masks the d–d band of Cu(ii) in complexes 1–3. The complexes 4–6 exhibited Cu-centered d–d band at ∼600 nm along with phenanthroline based π → π* and n → π* bands in the UV region (Fig. S13, ESI†). Curcumin complexes 1–3 showed an emission band with maximum at ∼520 nm on excitation at ∼440 nm in DMF at ambient temperature (Fig. 1). The emission of green light by the complexes enabled us to study their cellular localization by confocal microscopy. The complexes 4–6 displayed quasi-reversible cyclic voltammetric response in DMF–0.1 M TBAP ∼0.1 V *vs.* Ag/AgCl reference electrode due to the Cu(ii)–Cu(i) redox couple (Fig. S14, ESI†). However, the Cu(ii)–curcumin complexes (1–3) were redox inactive at the copper center in the usual potential window.^48^ Structural rigidity could be a probable reason for redox inactivity at the Cu(ii) center for 1–3. Therefore, the curcumin complexes are expected to be free from dark toxicity that arises from the copper-centered redox activity in cells due to the presence of reducing thiols such as glutathione.

**Table 1:** Selected physicochemical data for the complexes [Cu(cur)(L)(ClO_4_)] (1–3) and [Cu(acac)(L)(ClO_4_)] (4–6) (L = phen, 1, 4; dpq, 2, 5; dppz, 3, 6)

Complex	IR^a^/cm^−1^*ν*(C=O), *ν*(C=C), *ν*(ClO_4_^−^)	*λ*_max_ b/nm (*ε*/M^−1^ cm^−1^)	*E*_f_ c/V (Δ*E*_p_/mV)	*μ*_eff_ d/*μ*_B_	log *P*_o/w_
			Cu(ii)–Cu(i)		
1	1560; 1507; 1058	438 (15 200), 271 (17 200)	—	1.81	−0.525
2	1590; 1516; 1080	448 (28 400), 297 (29 600), 257 (46 400)	—	1.79	0.345
3	1586; 1511; 1073	438 (14 800), 384sh (11 600), 283 (27 600)	—	1.80	0.945
4	1585; 1514; 1081	616 (30), 443 (70), 271 (26 800)	0.03 (99)	1.78	−0.805
5	1566; 1514; 1097	621 (60), 336sh (3800), 293 (18 400), 257 (36 000)	0.11 (92)	1.79	−0.180
6	1571; 1520; 1077	606 (40), 437 (100), 377 (6400), 360 (6400), 275 (30 800)	0.13 (108)	1.79	0.013

a In KBr phase.

b In DMF–Tris–HCl buffer (1 : 4 v/v).

c Cu(ii)–Cu(i) couple in DMF–0.1 M TBAP, *E*_f_ = 0.5(*E*_pa_ + *E*_pc_), Δ*E*_p_ = (*E*_pa_ − *E*_pc_), where *E*_pa_ and *E*_pc_ are the anodic and cathodic peak potentials, respectively. The potentials are *vs.* Ag/AgCl electrode. Scan rate = 50 mV s^−1^.

d Magnetic moment at 298 K.

### 3.2 Solubility and stability

Complexes 1–6 were soluble in MeOH, MeCN, DMF, DMSO and their aqueous mixtures. All the complexes were insoluble in CHCl_3_, CH_2_Cl_2_ and hydrocarbon solvents. The stability of the curcumin complexes in solution phase was determined by monitoring their electronic absorption peak ∼440 nm. The UV-visible spectra of the complexes 1–3 recorded over a period of 24 h in DMSO–Tris–HCl buffer (1 : 4, pH = 7.2) (Fig. S15, ESI†) showed that they were significantly stable for a period of 24 h. However, curcumin is reported to undergo complete degradation in 24 h as evident from the absorption spectral traces.^49^ This clearly demonstrates the significant enhancement in stability of curcumin on ligation to Cu(ii) center along with the phenanthroline bases. To ascertain that the curcumin complexes do not undergo photo-degradation in visible light, we also recorded the UV-visible spectra of the complexes 1–3 every 10 min up to a period of 1 h keeping them exposed to visible light (400–700 nm). There were no significant spectral changes indicating that they do not undergo photo-bleaching in visible light in DMSO–Tris–HCl buffer (1 : 4, pH = 7.2) (Fig. S16, ESI†).

### 3.3 Crystal structure

**Fig. 2:**
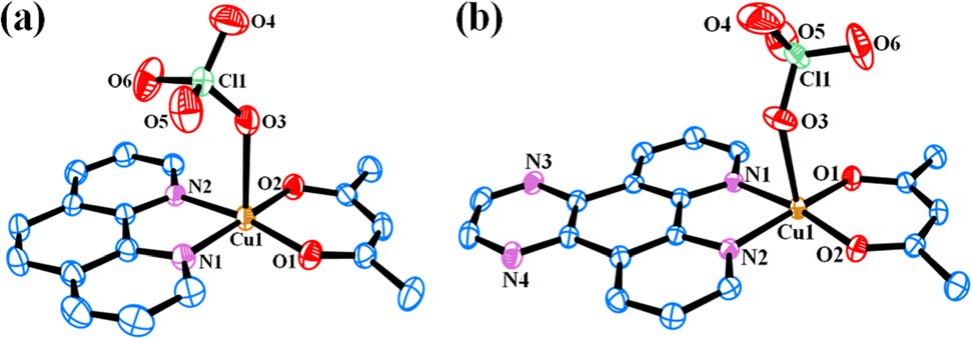
ORTEP views of complexes [Cu(acac)(phen)(ClO_4_)] (4) (a) and [Cu(acac)(dpq)(ClO_4_)] (5) (b) showing 50% probability thermal ellipsoids and the atom numbering scheme for the metal and hetero atoms. The hydrogen atoms are omitted for clarity.

Solid-state structures of the complexes 4 and 5 were determined by single crystal X-ray diffraction technique. The ORTEP views of the complexes are given in Fig. 2. Selected crystallographic data are shown in Table 2. Selected bond distance and angle data are given in Tables S1 and S2 (ESI†). Complex 4 crystallized in the *P*1̄ space group in triclinic crystal system with two molecules in the unit cell (Fig. S17, ESI†). The structure of 4 consists of a discrete complex having Cu(ii) displaying a slightly distorted square-pyramidal CuN_2_O_3_ geometry (degree of trigonality, *τ* = 0.026). Both acetylacetonato and 1,10-phenanthroline ligands coordinate in bidentate fashion with the Cu–O and Cu–N bond distances in the range 1.898(18) to 2.414(2) Å and 1.999(2) to 2.008(2) Å respectively. The perchlorate anion coordinates from the axial position with Cu–O bond distances of 2.414(2) Å. Complex 5 crystallized in the *P*21/*n* space group in monoclinic crystal system with four molecules in the unit cell (Fig. S18, ESI†). It has similar coordination environment as 4 with slightly distorted square-pyramidal CuN_2_O_3_ geometry (*τ* = 0.015). The Cu–O and Cu–N bond distances in the range 1.898(3) to 2.402(3) Å and 2.005(3) to 2.017(3) Å respectively. The Cu–O bond distance for the weakly coordinated perchlorate anion is 2.402(3) Å. Similar crystal structures with different anions such as chloride or nitrate and solvents of crystallization were reported earlier.^50,51^ However, they crystallized in different space groups or crystal systems.

**Table 2:** Selected crystallographic data for the complexes [Cu(acac)(phen)(ClO_4_)] (4) and [Cu(acac)(dpq)(ClO_4_)] (5)

Empirical formula	C_17_H_15_ClCuN_2_O_6.50_	C_19_H_15_ClCuN_4_O_6_
*F*_w_, g M^−1^	450.30	494.34
Crystal system	Triclinic	Monoclinic
Space group	*P*1̄	*P*21/*n*
*a*, Å	8.596(2)	10.1600(12)
*b*, Å	10.119(2)	9.5194(10)
*c*, Å	12.532(3)	20.454(3)
*α*, °	97.262(11)	90
*β*, °	105.219(11)	102.925(7)
*γ*, °	111.170(11)	90
*V*, Å^3^	950.7(4)	1928.1(4)
*Z*	2	4
*T*, K	296(2)	296(2)
*ρ*_calcd_, g cm^−3^	1.573	1.703
*λ*, Å (Mo-K_α_)	0.71073	0.71073
*μ*, cm^−1^	1.328	1.319
Data/restraints/parameters	5952/0/252	4514/0/283
*F*(000)	458	1004
Goodness-of-fit	1.082	1.081
*R*(*F*_o_)^a^, *I* > 2*σ*(*I*) [w*R*(*F*_o_)^b^]	0.0463 [0.1439]	0.0559 [0.1479]
*R* (all data) [w*R* (all data)]	0.0671 [0.1647]	0.0830 [0.1649]
Largest diff. peak and hole (e Å^−3^)	0.982, −0.706	1.624, −0.678

a *R* = Σ‖*F*_o_| − |*F*_c_‖/Σ|*F*_o_|.

b w*R* = {Σ[*w*(*F*_o_^2^ − *F*_c_^2^)^2^]/Σ[*w*(*F*_o_)^2^]}^1/2^; *w* = [*σ*^2^(*F*_o_)^2^ + (*AP*)^2^ + *BP*]^−1^, where *P* = (*F*_o_^2^ + 2*F*_c_^2^)/3, *A* = 0.1020; *B* = 0.0068 for 4 and *A* = 0.0861; *B* = 1.0893 for 5.

### 3.4 DNA and protein binding property

DNA is a major target of many anticancer drugs including cisplatin that forms covalent adduct with DNA and Fe(ii)–bleomycins that exhibit non-covalent interaction. The ability of the complexes 1–6 to bind to calf-thymus (CT) DNA was evaluated by UV-visible titration method. The intrinsic DNA binding constant (*K*_b_) of 1–6 were determined by monitoring the change in the absorption at the ligand-centered band of the complexes 1–6 at ∼260–270 nm. The absorption titration revealed significant hypochromicity along with minor bathochromic shifts which suggests primarily groove binding mode of the complexes to CT-DNA (Fig. S19, ESI†). Classical DNA intercalators such as ethidium bromide π-stack between two DNA base pairs and cause much larger hypochromicity and significant bathochromic shift of the absorption bands.^52^ The *K*_b_ values of the complexes determined using McGhee and von Hippel equation fall in the range (3.18 ± 0.19) × 10^5^ to (9.85 ± 0.50) × 10^5^ M^−1^ giving the DNA binding affinity order as: 3 > 2 > 1 and 6 > 5 > 4 (Table S3, ESI†). The complexes 3 and 6 having the extended aromatic phenazine ring in their formulations probably partially intercalates between DNA bases through the DNA groove resulting in their higher binding constants compared to other compounds. The relatively higher binding constants observed for 3 compared to 6 could be the result of H-bonding interactions of the curcumin moiety to the DNA backbone.

The binding affinity of the complexes 1–6 to human serum albumin (HSA) was assessed by tryptophan emission-quenching experiment.^53,54^ The intensity of emission of HSA in Tris–HCl buffer (pH = 7.2) at ∼320 nm decreased gradually with increasing concentration of 1–6 due to their interaction with the protein molecules (Fig. S20, ESI†). The plots of *I*_0_/*I vs.* [complex] were found to be linear and slopes gave the *K*_HSA_ values in an order 3 > 2 > 1 and 6 > 5 > 4 (Table S3, ESI†). Fluorescence lifetime of neat HSA and in presence of two different concentrations of complex 3 (10 and 20 μM) were recorded to find out whether quenching happens through excited state interaction or through ground state complexation of the complexes with HSA. The experiment has revealed that the quenching mechanism is static in nature, which happens through ground-state coordination of the complexes to HSA (Fig. S21, ESI†). The higher binding affinity of the complexes 3 and 6 indicate that the presence of the planar hydrophobic dipyridophenazine ligand assists the binding of the complexes probably to some hydrophobic pocket of the serum protein. The curcumin complexes exhibited relatively higher binding propensity for HSA compared to their acetylacetonato analogues probably due to hydrophobic and/or H-bonding interactions mediated by curcumin. This moderate binding propensity implies that human serum albumin could be potentially carrier protein for Cu(ii) complexes.^55,56^

### 3.5 Photocytotoxicity study

**Fig. 3:**
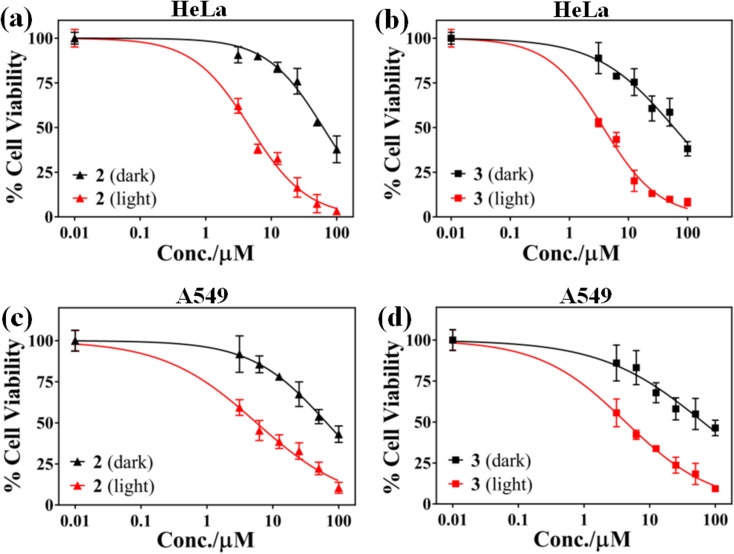
Cell viability plots showing the cytotoxic effect of complexes 2 and 3 in HeLa (a & b) and A549 (c & d) cells in dark (black symbols) and in the presence of visible light (red symbols, 400–700 nm, 10 J cm^−2^, 1 h).

*In vitro* photo-enhanced toxicity of the curcumin complexes 1–3 against human cervical carcinoma (HeLa) and adenocarcinomic human alveolar basal epithelial (A549) cells were evaluated using MTT [3-(4,5-dimethylthiazol-2-yl)-2,5-diphenyltetrazolium bromide] assay and compared to those of the control complexes 4–6 (Fig. 3 and S22–S25, ESI†). The complexes 1–3 displayed remarkable dose dependent photocytotoxicity in visible light (400–700 nm) in both HeLa and A549 cells while they were largely non-toxic in dark. An 8–16 fold enhancement in cytotoxicity is observed for the complexes 1–3 in HeLa cells for a light exposure of 1 h. The complexes 4–6 without curcumin were not cytotoxic in dark as well as light exposure. Complex 3 was found to be most active in killing HeLa cells on visible light irradiation with IC_50_ value of 3.7 μM in light. This is comparable to the activity of the commercially available drug Photofrin®. A similar pattern of antiproliferative activity was observed in A549 cells. Complex 3 was most active in A549 cells and displayed lowest IC_50_ value of 4.3 μM under similar conditions on exposure to low energy visible light. The low dark toxicity of the curcumin complexes is probably the result of redox inactivity at the copper center, which otherwise generates cytotoxic ROS under the action of cellular thiols. The control complexes 4–6 were probably photo-inactive or unable to enter the cells and hence were non-toxic. A comparison of the IC_50_ values of the copper complexes studied, Photofrin® and curcumin are presented in Table 3.^57–60^ The phenanthroline bases and the metal salt were found to be non-toxic in dark and visible light in both the cancer cell lines.

**Table 3:** The IC_50_ values of Photofrin®, curcumin and [Cu(cur)(L)(ClO_4_)] (1–3) and [Cu(acac)(L)(ClO_4_)] (4–6) (L = phen, 1, 4; dpq, 2, 5; dppz, 3, 6) in HeLa and A549 cells

Compound	IC_50_(μM)					
	HeLa			A549		
	Dark^a^	Light^b^	PI^c^	Dark^a^	Light^b^	PI^c^
1	56.4 ± 3.5	6.8 ± 0.9	8.3	85.3 ± 5.4	8.6 ± 0.8	9.9
2	61.9 ± 3.9	4.7 ± 0.8	13.2	65.6 ± 4.4	5.7 ± 0.7	11.5
3	58.9 ± 3.7	3.7 ± 0.5	15.9	64.9 ± 4.3	4.3 ± 0.4	15.1
4	>100	41.0 ± 3.8	>2.4	>100	48.8 ± 4.2	>2.0
5	>100	46.8 ± 3.9	>2.1	>100	45.1 ± 3.5	>2.2
6	>100	36.9 ± 2.8	>2.7	>100	39.8 ± 3.3	>2.5
Photofrin®	>41^d^	4.3 ± 0.2^d^	>9.5	>50^e^	0.50 ± 0.04^e^	>100
Curcumin	85.4 ± 0.6^f^	8.2 ± 0.2^f^	10.4	61.4 ± 0.4^g^	19.0 ± 0.1^g^	3.2

a The IC_50_ values correspond to 4 h treatment followed by 20 h incubation in dark.

b The IC_50_ values correspond to 4 h treatment in dark followed by photo-exposure to visible light (400–700 nm, 10 J cm^−2^) for 1 h. Subsequently, cells were incubated for 19 h in dark.

c PI (Phototoxic Index) is the ratio between the IC_50_ values in the dark and visible light exposure.

d The Photofrin® IC_50_ values (633 nm excitation; fluence rate: 5 J cm^−2^) are taken from ref. 59 (converted to μM using the approximate molecular weight of Photofrin®, 600 g M^−1^).

e The IC_50_ values are taken from ref. 60.

f The IC_50_ values are taken from ref. 58.

g The IC_50_ values are taken from ref. 57.

### 3.6 Measurement of lipophilicity and cellular uptake

Lipophilicity is considered as a primary physicochemical property of low molecular weight anticancer drugs for effective extravascular transport.^61^ We estimated the partition coefficients of 1–6 between *n*-octanol and water (expressed as log *P*_o/w_) to find out if there were any correlation between lipophilicity and cytotoxicity of the compounds. The log *P*_o/w_ values were found to follow an order: 3 > 2 > 1 and 6 > 5 > 4 (Table 1). The Cu(ii)–curcumin complexes were more lipophilic compared to their acetylacetonato analogues. The complexes showed an increase in lipophilicity with extended aromatic rings of the phenanthroline bases. The Cu(ii) curcumin complex 3 with dipyridophenazine ligand was the most lipophilic with log *P*_o/w_ value of 0.945. The copper acetylacetonato complexes 4–6 were hydrophilic in nature with negative log *P*_o/w_ values. The curcumin complexes 1–3 were highly photocytotoxic probably due to their enhanced uptake in cancer cells owing to their higher lipophilicity as well as higher photosensitizing ability in visible light. However, the redox inactivity at the Cu(ii) centre of 1–3 make them virtually nontoxic in dark resulting in high photocytotoxicity indices.

Preliminary study on the uptake of the green fluorescent curcumin appended complexes (1–3) in HeLa cells was carried out with the help flow cytometry.^62^ HeLa cells treated with the complexes 1–3 (10 μM) for 4 h were analysed to determine their uptake into the cells. There were significant enhancement in the fluorescence of the complex treated cells compared to cells' auto fluorescence indicating substantial uptake of the lipophilic Cu(ii) curcumin complexes in HeLa cells in 4 h (Fig. S26, ESI†). The complex 3 having dppz ligand along with curcumin was found to have highest uptake in the time period of study probably due to its highest lipophilicity of the lot. The order of uptake of 1–3 in 4 h follows the order of their lipophilicty as evident from flow cytometry experiment.

### 3.7 Cellular ROS generation

**Fig. 4:**
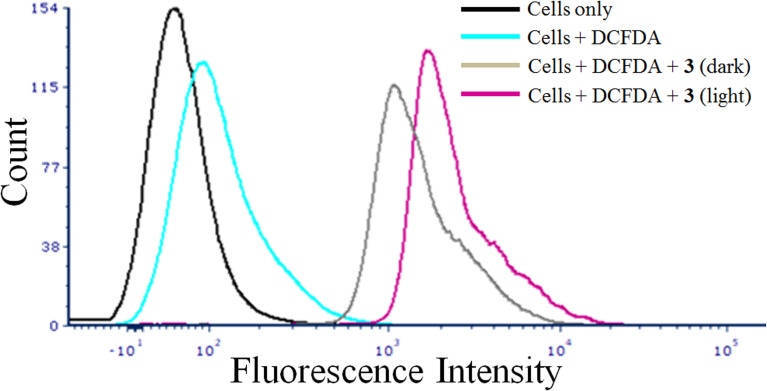
Flow cytometry to detect the ROS generation in HeLa cells using DCFDA assay for the complex 3 with different experimental conditions shown in color codes: cells only (black); cells + DCFDA (cyan); cells + DCFDA + complex 3 (in dark) (grey); and cells + DCFDA + complex 3 (in light) (magenta). A greater shift in fluorescence intensity implies higher amount of DCF and greater ROS generation.

DCFDA assay was employed to evaluate the ability of the complexes to generate reactive oxygen species in cancer cells. We have used complex 3 as a representative complex for this assay. H_2_DCF, formed by enzymatic reaction from DCF, is converted to DCF on oxidation by intracellular ROS.^63^ The emissive property of DCF in green light (*λ*_em_ = 528 nm) was used to monitor the ability of 3 to generate ROS in HeLa cells by using flow cytometry. A significant increase in emission by DCF formed by intracellular oxidation of DCFDA by ROS generated from the photo-excitation of the complex 3 was observed (Fig. 4). This was evident by the significant shift of the emission band of DCF towards right in the light exposed and compound treated HeLa cells. The control experiments with only cells in dark, light and compound treated cells kept in dark did not show any significant ROS formation in the dark. The ROS generated by the complexes on light exposure is believed to be primarily responsible of the cell death. The acetylacetonato complexes did not show any significant generation of ROS in both dark and visible light under similar experimental conditions.

### 3.8 Flow cytometric analysis for mechanism of cell death

**Fig. 5:**
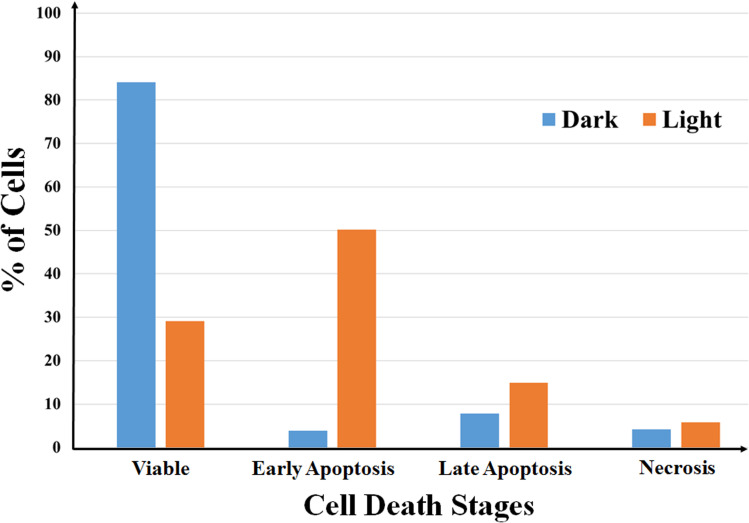
A bar diagram showing annexin V-FITC-PI staining of HeLa cells undergoing apoptosis induced by complex 3 (5 μM) in dark and visible light (400–700 nm, 10 J cm^−2^) analyzed by flow cytometry.

Annexin V-FITC-PI assay was used to quantify number of cells undergoing apoptosis. In flow cytometry, annexin V is commonly used to detect apoptotic cells, as it is able to bind to phosphatidylserine, a marker of apoptosis on the outer membrane of cells due to membrane flipping.^64^ The annexin V-FITC-PI assay was performed on 1 and 3 treated HeLa cells both in dark and light (400–700 nm) and the results were analyzed using fluorescence assisted cell sorting (FACS) analysis (Fig. 5, S27 and S28, ESI†). The assay gives an estimate of early or late apoptotic, necrotic and viable cell population. Fig. 5 shows the population of HeLa cells in all the four stages of cell cycle treated with complex 3 in both dark and light. From the figure it is evident that complex 3 is capable of inducing apoptosis in HeLa cells as the percentage of cell population in the apoptotic stage increases to a significant extent on exposure to visible light. The population of cells in early apoptotic stage increased from ∼4% in dark to ∼50% on visible light exposure, whereas the population of cells in late apoptotic stage increased from ∼8% in dark to ∼15% during the same experiment. Complex 1 also showed visible light assisted induction of apoptosis in HeLa cells, however not up to the extent of 3 (Fig. S27, ESI†). However, no such increase in population of cells undergoing apoptosis was observed in the control experiments. These observations indicate that the complexes kill cancer cells primarily *via* apoptosis mechanism on photo-irradiation.

### 3.9 Confocal imaging

**Fig. 6:**
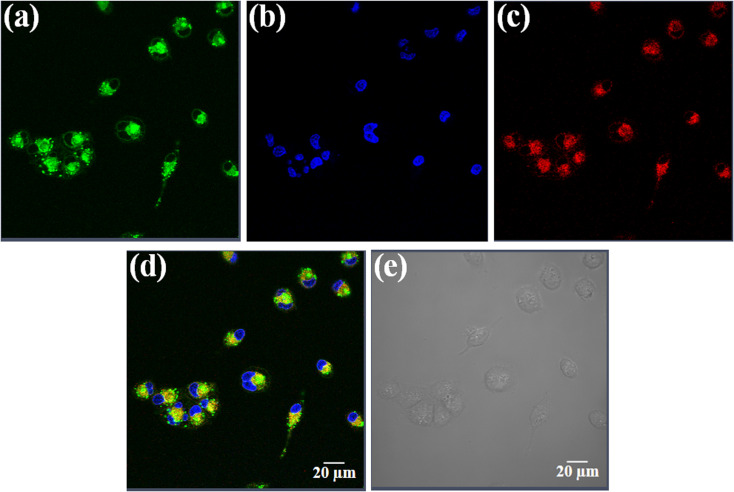
Confocal microscopic images of HeLa cells treated with complex 3 (10 μM) for 4 h and ER-tracker red and 4′,6-diamidino-2-phenylindole (DAPI). Panel (a) corresponds to the green emission of the curcumin complex 3. Panel (b) corresponds to the blue emission of DAPI. Panel (c) corresponds to the red emission of ER-tracker red. Panels (d) is the merged images of the first three panels and (e) is the bright field image. Scale bar = 20 μm.

Uptake and localization of anticancer agents in specific organelles of the cell is key for their activity.^65^ Complex 3 having the fluorescent curcumin ligand was used for cellular imaging using confocal microscopy in HeLa cells (Fig. 6, panels a–e). The cells were co-stained with the nuclear staining dye 4′,6-diamidino-2-phenylindole (DAPI), which emits blue fluorescence (Fig. 6, panel b). Complex 3 (10 μM) showed predominant cytosolic localization in HeLa cells on treatment for a time span of 4 h as evident from the curcumin based green fluorescence (Fig. 6, panel a). To find whether the complexes display any specific sub-cellular localization, the HeLa cells were co-stained with ER-tracker that emits red fluorescence (Fig. 6, panel c). The merged images clearly show that the curcumin complexes predominantly localizes in the endoplasmic reticulum of HeLa cells (Fig. 6, panel d). The bright field image is shown in panel e (Fig. 6).

## 4. Conclusion

Three copper(ii) complexes having curcumin and phenanthroline bases were synthesized, fully characterized and are evaluated for photo-activated anticancer activities. The corresponding copper complexes having acetylacetonato ligand in place of curcumin were synthesized to compare the efficacies of the curcumin complexes. The chelation to copper enhances the stability of curcumin at physiological pH and improves its antitumor activity upon photo-activation. The phenanthroline bases present in their structures assist the dual function of photosensitizer cum binder to biomolecules. The complexes display efficient binding efficacy to calf thymus DNA probably through partial intercalation between DNA bases. The moderate binding propensity to human serum albumin implies that the complexes could be transported in blood *via* binding to serum albumin. The complexes are excellent photo-cytotoxic agents in HeLa and A549 cancer cells. They are capable of generating ROS in cancer cells on exposure to low energy visible light. The curcumin complexes localize in endoplasmic reticulum of HeLa cells. The work presented in this article highlights the scope of development of simple curcumin based first row transition metal complexes with excellent photo-enhanced cytotoxicity in cancer cells.

## Author contributions

Atrayee Banaspati: conceptualization, data curation, formal analysis, investigation, methodology, writing-original draft. Vanitha Ramu: data curation, formal analysis, investigation, methodology. Md Kausar Raza: conceptualization, data curation, formal analysis, investigation, methodology, supervision, validation, visualization, writing-review & editing. Tridib K. Goswami: conceptualization, data curation, formal analysis, investigation, methodology, funding acquisition, project administration, supervision, validation, visualization, writing-review & editing.

## Conflicts of interest

There is no conflict of interest to declare.

## Supplementary Material

RA-012-D2RA04813B-s001

RA-012-D2RA04813B-s002
