# Supplementary material for: Copper(ii) curcumin complexes for endoplasmic reticulum targeted photocytotoxicity
Source: RSC Adv. 2022 Oct 27;12(47):30722–33. doi: 10.1039/d2ra04813b (PMC9606729; doi:10.1039/d2ra04813b)
Supplement: RA-012-D2RA04813B-s001 [file RA-012-D2RA04813B-s001.pdf]

Electronic Supplementary Material (ESI) for RSC Advances.
This journal is © The Royal Society of Chemistry 2022

Supporting Information for

Copper(II) Curcumin Complexes for Endoplasmic Reticulum Targeted Photocytotoxicity

Atrayee Banaspati,[†] Vanitha Ramu,[b] Md Kausar Raza,*[,‡] and Tridib K. Goswami*[,†]

[†]*Department of Chemistry, Gauhati University, Guwahati 781014, Assam, India*

[‡]*Department of Inorganic and Physical Chemistry, Indian Institute of Science, Bangalore 560012, India*

*Corresponding author. *E-mail address*: tridibgoswami05@gmail.com (T. K. Goswami); kausarraza91@gmail.com (M. K. Raza).

Table of Contents

Table S1. Selected bond distances (Å) and bond angles (^{0}) of [Cu(acac)(phen)(ClO_4)] (**4**).

Table S2. Selected bond distances (Å) and bond angles (^{0}) of [Cu(acac)(dpq)(ClO_4)] (**5**).

Table S3. DNA and protein binding constants for the Complexes [Cu(Cur)(L)(ClO_4)] (**1-3**) and [Cu(acac)(L)(ClO_4)] (**4-6**) (L = phen, **1, 4**; dpq, **2, 5**; dppz, **3, 6**).

Scheme S1. Synthetic scheme for the complexes **1**-**3**.

Scheme S2. Synthetic scheme for the complexes **4**-**6**.

Figures S1-S6. ESI-MS spectra of complex **1**-**6** in methanol.

Figures S7-S12. IR spectra of the complexes **1-6** in solid KBr matrix.

Figure S13. The electronic spectra of the complexes **1**-**3** (a) and **4**-**6** (b) (1 mM) in DMF-Tris-HCl buffer (pH 7.2) (1:4 v/v).

Figure S14. Cyclic voltammograms of complexes **3-6** in DMF using TBAP (0.1 M) as the supporting electrolyte with a scan rate of 50 mV s^{-1}.

Figure S15. Electronic absorption spectra of the complexes **1-3** (a-c) in DMSO-Tris-HCl buffer (pH 7.2) (1:4 v/v) at different time points showing the solution phase stability of the complexes.

Figure S16. Electronic absorption spectra of the complexes **1** (a), **2** (b) and **3** (c) in DMSO-Tris-HCl buffer (pH 7.2) (1:4 v/v) at different time points of exposure to visible light (400-700 nm).

Figure S17. The unit cell packing diagram of [Cu(acac)(phen)(ClO_4)] (**4**) having two molecules in the unit cell.

Figure S18. The unit cell packing diagram of [Cu(acac)(dpq)(ClO_4)] (**5**) having four molecules in the unit cell.

Figure S19. Absorption spectral traces of the complexes **1-6** (**a-f**) in 5 mM Tris-HCl buffer (pH 7.2) on increasing the quantity of calf thymus DNA. The inset shows the least square fits of $\Delta\varepsilon_{af}/\Delta\varepsilon_{bf}$ vs. [DNA] for the complexes using McGhee-von Hippel (MvH) method.

Figure S20. Emission spectral traces of HSA (2 μM) in the presence of complexes **1-6** (**a-f**). The inset shows the plot of (I_0/I) vs. [complex].

Figure S21. Fluorescence intensity decay profiles of HSA in the absence and presence of 10 and 20 μM of complex **3** in phosphate buffer (pH 7.4).

Figures S22-S25. Cell viability plots showing the cytotoxic effect of complexes **1**, **4-6** in HeLa and A549 cells in dark (black symbols) and in the presence of visible light (red symbols, 400-700 nm, 10 J cm^{-2}, 1 h).

Figure S26. Uptake of the complexes **1**-**3** (10 μM) at 37 °C for 4 h incubation time in HeLa cells determined by flow cytometry.

Figure S27. Annexin V-FITC-PI staining of HeLa cells undergoing apoptosis induced by complex **1** (5 μM) in dark and visible light (400-700 nm, 10 J cm^{-2}) analyzed by flow cytometry.

Figure S28. Annexin V-FITC-PI staining of HeLa cells undergoing apoptosis induced by complex **3** (5 μM) in dark and visible light (400-700 nm, 10 J cm^{-2}) analyzed by flow cytometry

Table S1. Selected bond distances (Å) and bond angles (^{0}) of [Cu(acac)(phen)(ClO_4)] (**4**)

Cu1-O1	1.8981(18)	O1-Cu1-N1	90.66(8)
Cu1-O2	1.9083(19)	O2-Cu1-N1	172.53(8)
Cu1-O3	2.414(2)	N2-Cu1-N1	82.41(8)
Cu1-N1	2.008(2)	O1-Cu1-O3	97.08(9)
Cu1-N2	1.999(2)	O2-Cu1-O3	89.15(8)
O1-Cu1-O2	94.47(8)	N2-Cu1-O3	90.57(8)
O1-Cu1-N2	170.16(8)	N1-Cu1-O3	95.61(8)
O2-Cu1-N2	91.81(8)		

Table S2. Selected bond distances (Å) and bond angles (^{0}) of [Cu(acac)(dpq)(ClO_4)] (**5**)

Cu1-N1	2.005(3)	O2-Cu1-N2	91.61(12)
Cu1-N2	2.017(3)	O1-Cu1-N2	168.73(13)
Cu1-O1	1.898(3)	N1-Cu1-N2	81.84(12)
Cu1-O2	1.895(3)	O2-Cu1-O3	99.22(13)
Cu1-O3	2.402(3)	O1-Cu1-O3	100.22(12)
O2-Cu1-O1	94.76(11)	N1-Cu1-O3	88.02(13)
O2-Cu1-N1	170.08(12)	N2-Cu1-O3	87.90(11)
O1-Cu1-N1	90.57(12)		

Table S3. DNA and protein binding constants for the Complexes [Cu(Cur)(L)(ClO_4)] (**1**-**3**) and [Cu(acac)(L)(ClO_4)] (**4**-**6**) (L = phen, **1, 4**; dpq, **2, 5**; dppz, **3, 6**).

Complex No.	Binding constant (M^{-1})	
	ct-DNA	HSA
**1**	7.69 (±0.39) × 10^5	1.67 (±0.02) × 10^5
**2**	8.42 (±0.33) × 10^5	3.11 (±0.03) × 10^5
**3**	9.85 (±0.50) × 10^5	3.20 (±0.02) × 10^5
**4**	3.18 (±0.19) × 10^5	1.16 (±0.01) × 10^5
**5**	5.96 (±0.54) × 10^5	1.73 (±0.03) × 10^5
**6**	8.73 (±0.43) × 10^5	2.95 (±0.02) × 10^5

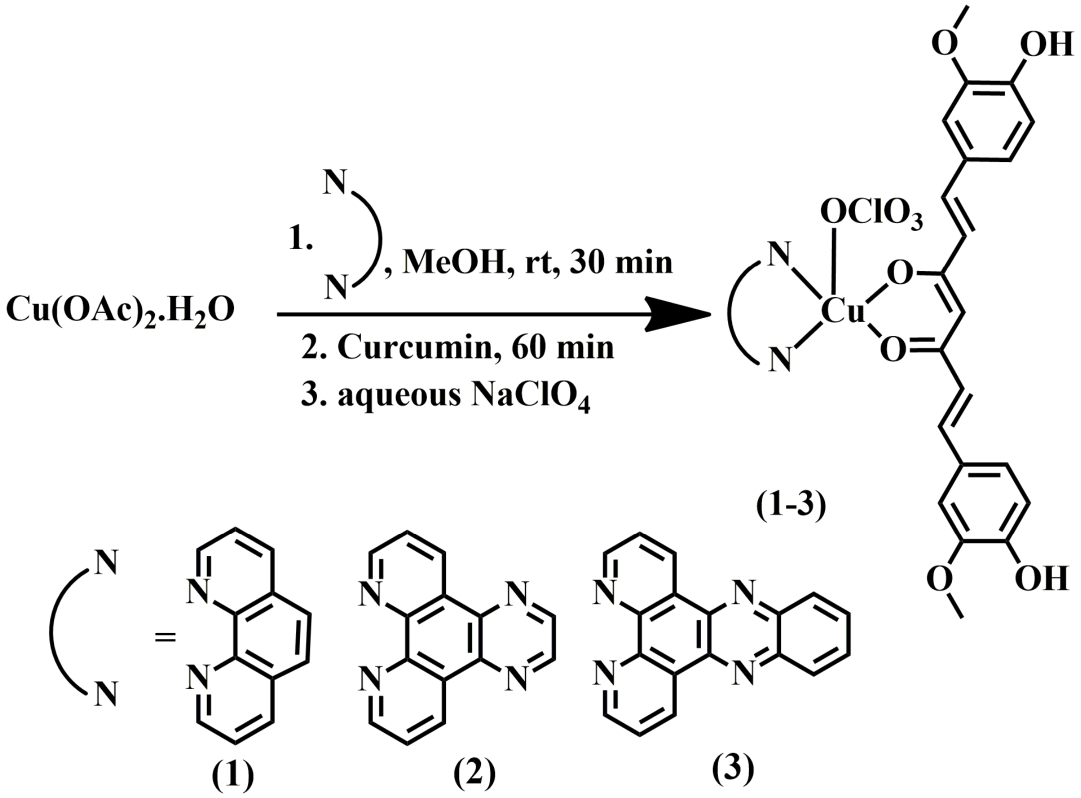

Scheme S1. Synthetic scheme for the complexes **1**-**3**.

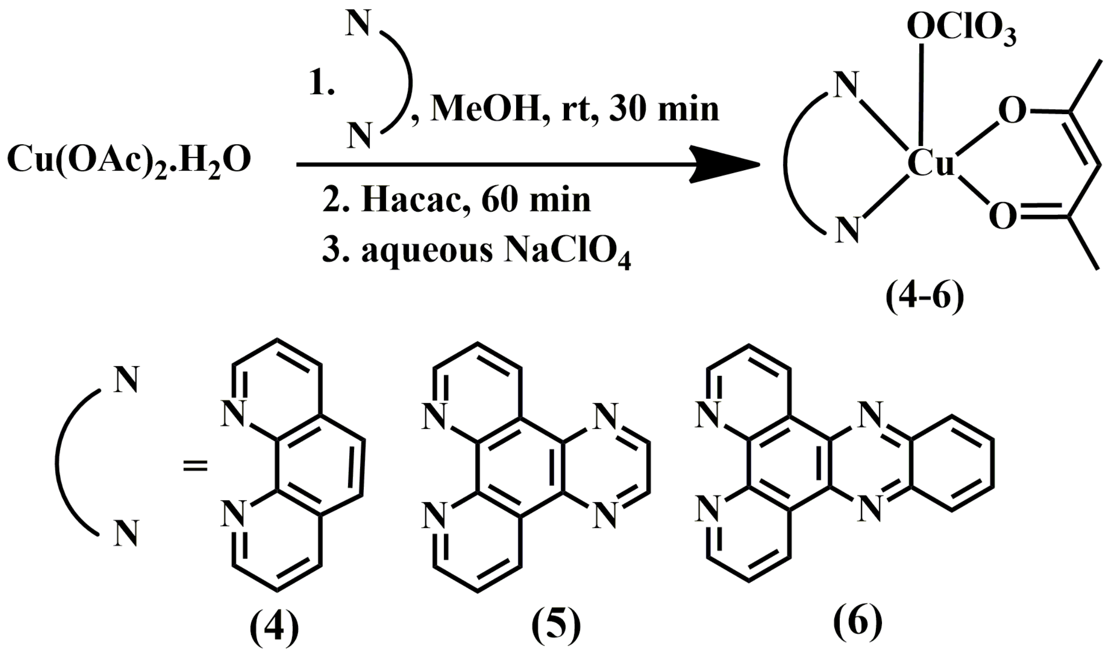

Scheme S2. Synthetic scheme for the complexes **4-6**.

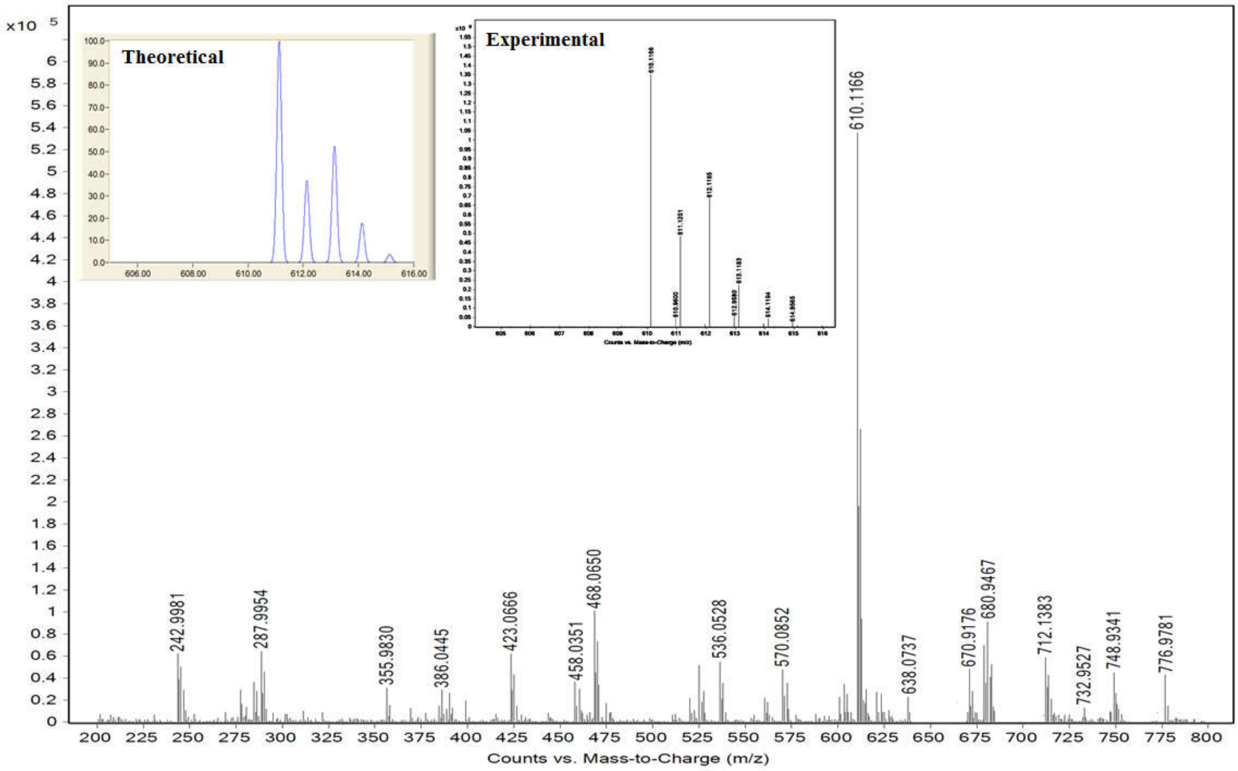

Figure S1. ESI-MS spectrum of complex **1** in methanol showing the $[M-(ClO_4^-)]^+$ peak at m/z = 610.1166. The inset shows the theoretical and experimental isotopic distributions for the complex.

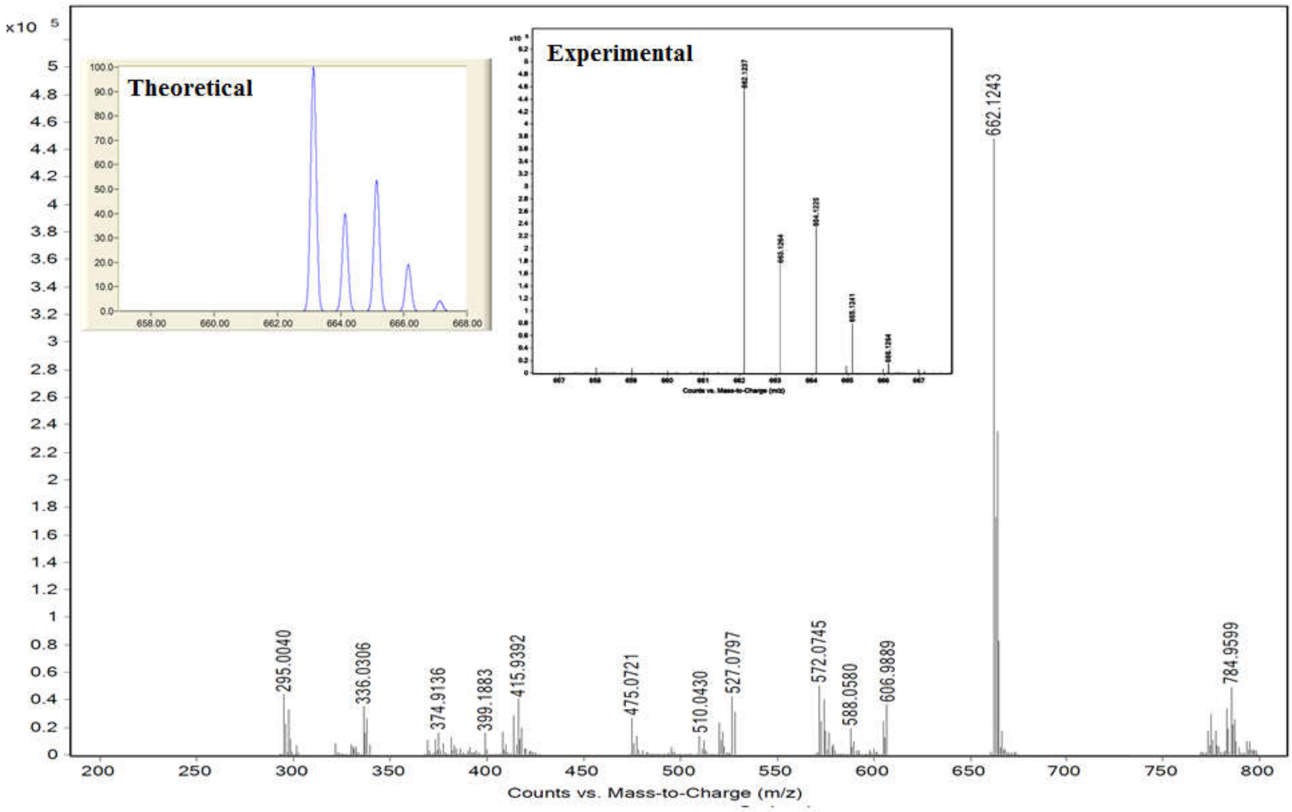

Figure S2. ESI-MS spectrum of complex **2** in methanol showing the [M-(ClO_4^-)]$^+$ peak at m/z = 662.1243. The inset shows the theoretical and experimental isotopic distributions for the complex.

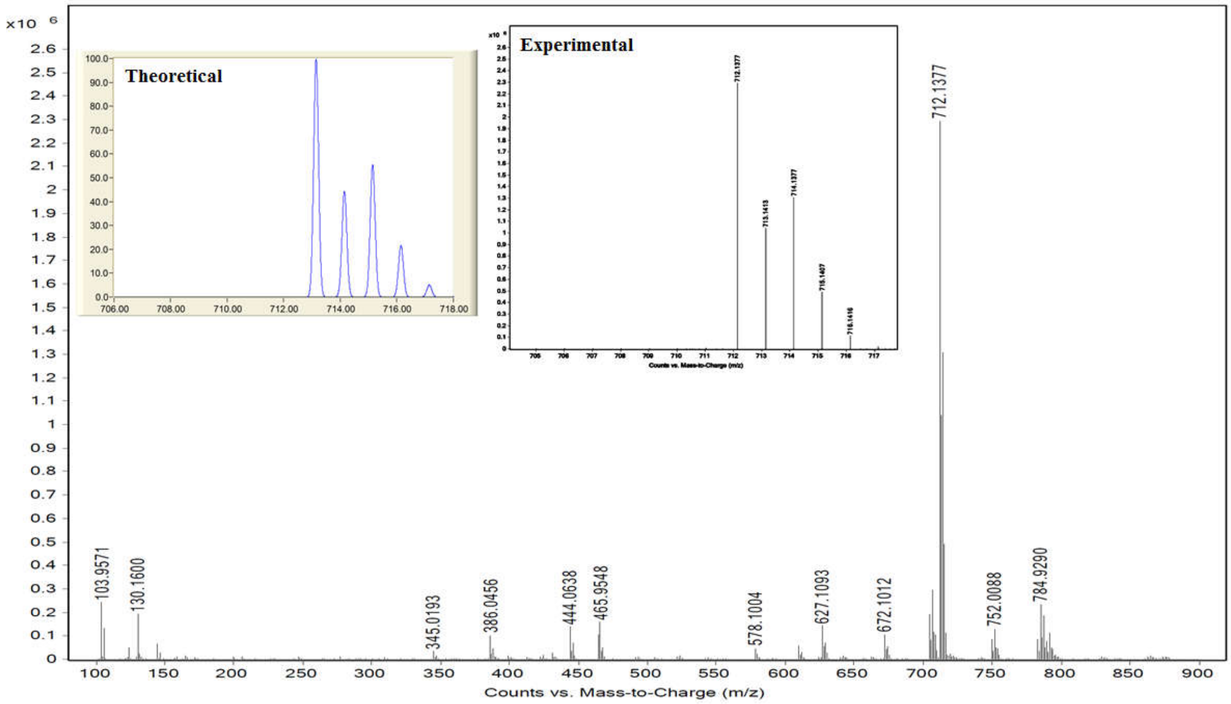

Figure S3. ESI-MS spectrum of complex **3** in methanol showing the $[M\text{-}(ClO_4^-)]^+$ peak at m/z = 712.1377. The inset shows the theoretical and experimental isotopic distributions for the complex.

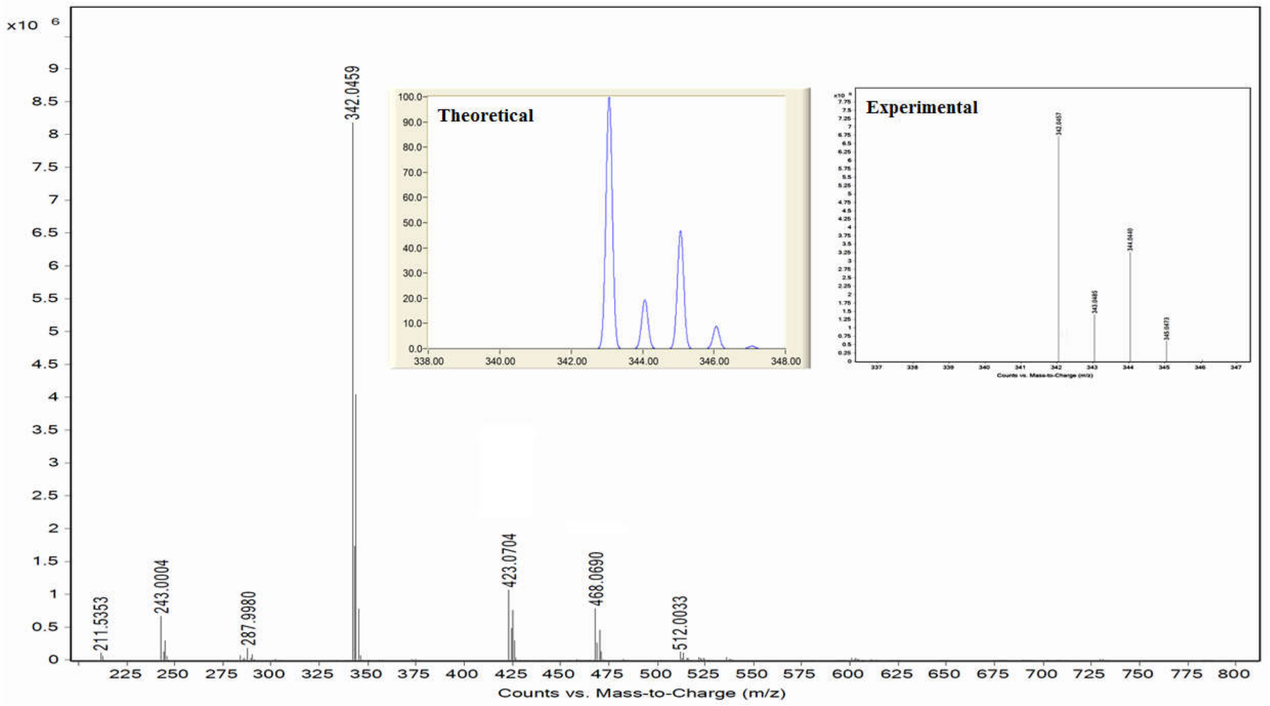

Figure S4. ESI-MS spectrum of complex **4** in methanol showing the $[M-(ClO_4^-)]^+$ peak at m/z = 342.0459. The inset shows the theoretical and experimental isotopic distributions for the complex.

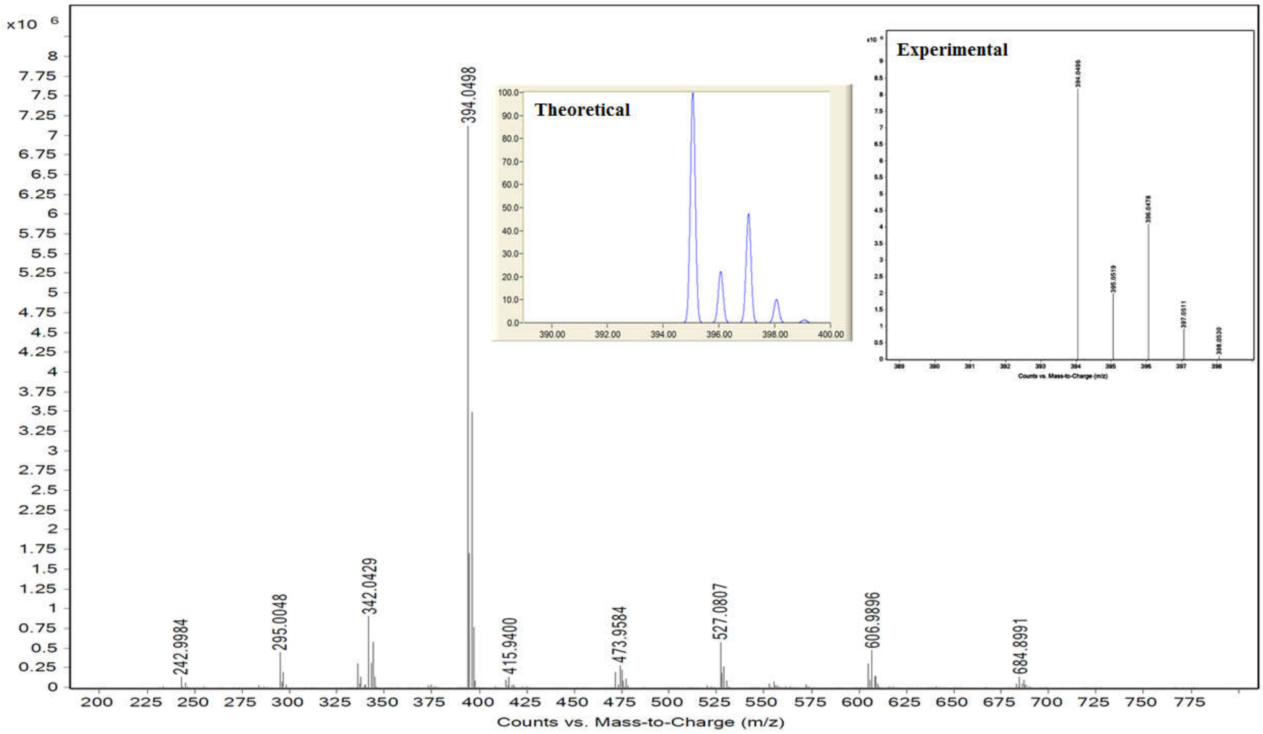

Figure S5. ESI-MS spectrum of complex **5** in methanol showing the $[M-(ClO_4^-)]^+$ peak at m/z = 394.0498. The inset shows the theoretical and experimental isotopic distributions for the complex.

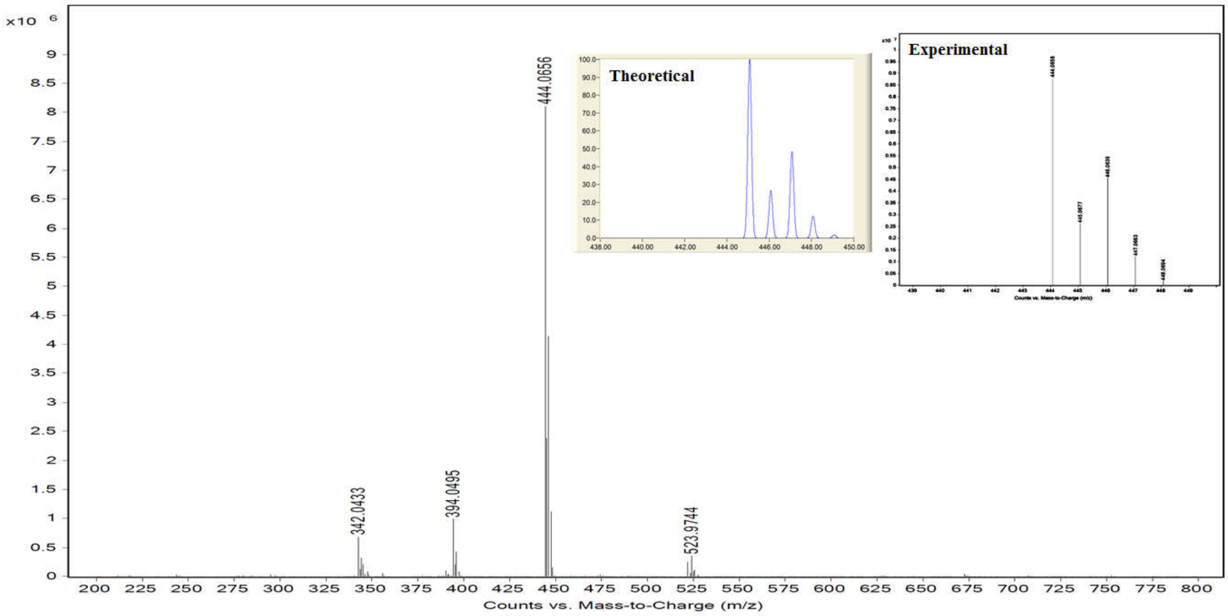

Figure S6. ESI-MS spectrum of complex **6** in methanol showing the $[M-(ClO_4^-)]^+$ peak at m/z = 444.0656. The inset shows the theoretical and experimental isotopic distributions for the complex.

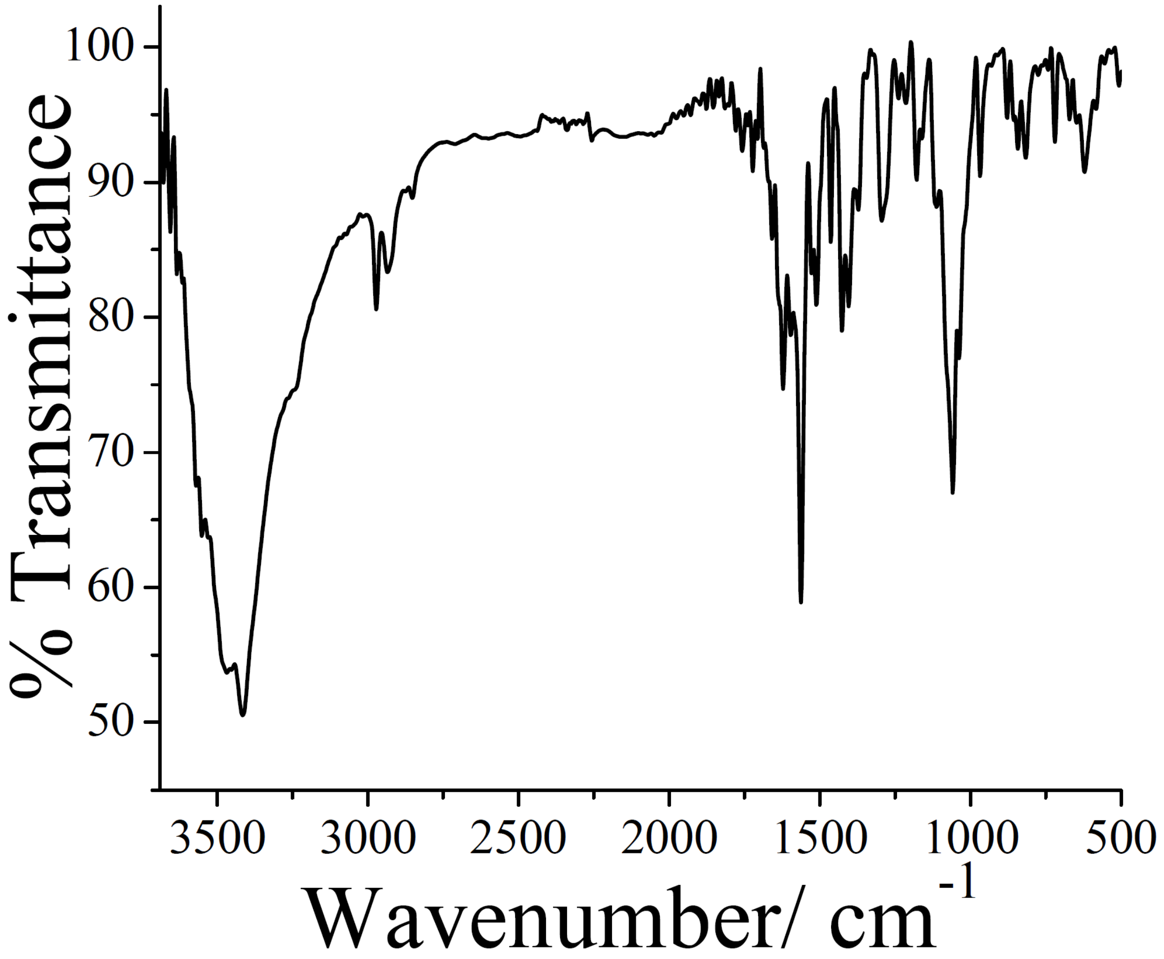

Figure S7. IR spectrum of complex **1**.

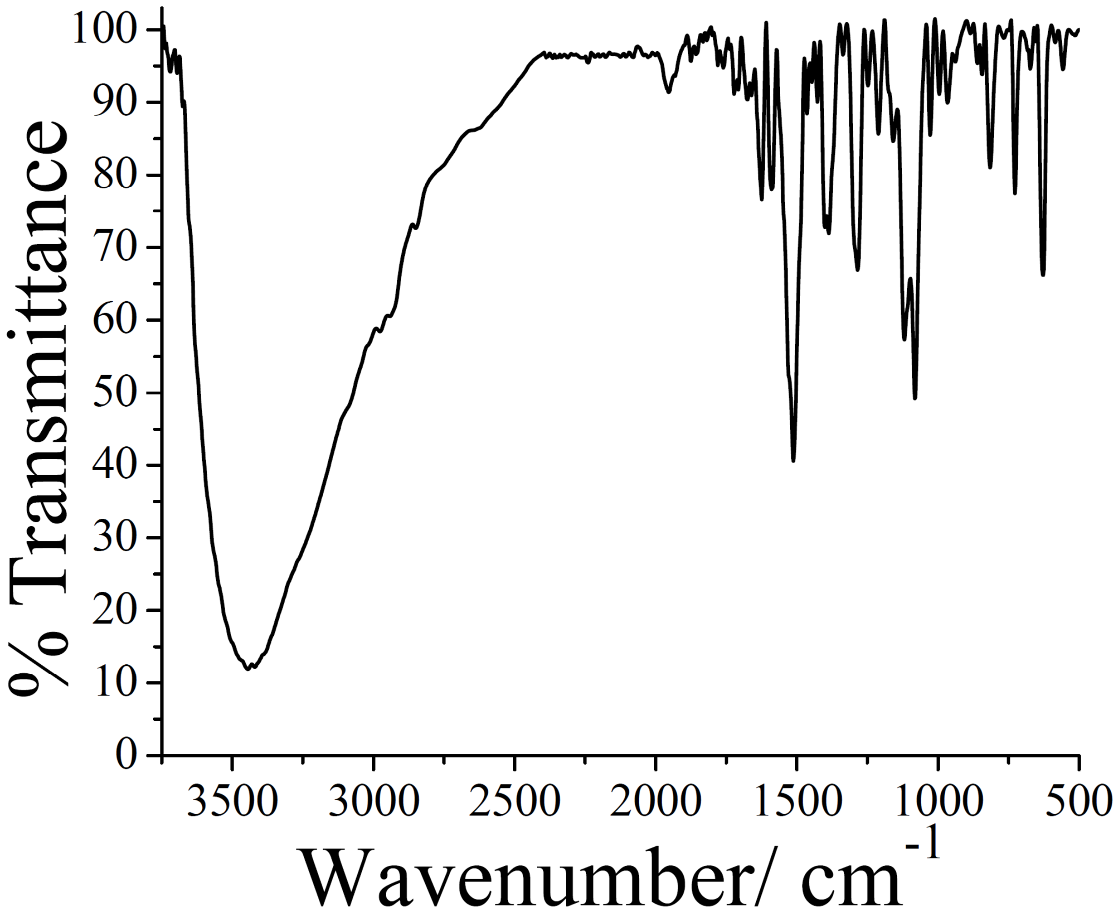

Figure S8. IR spectrum of complex **2**.

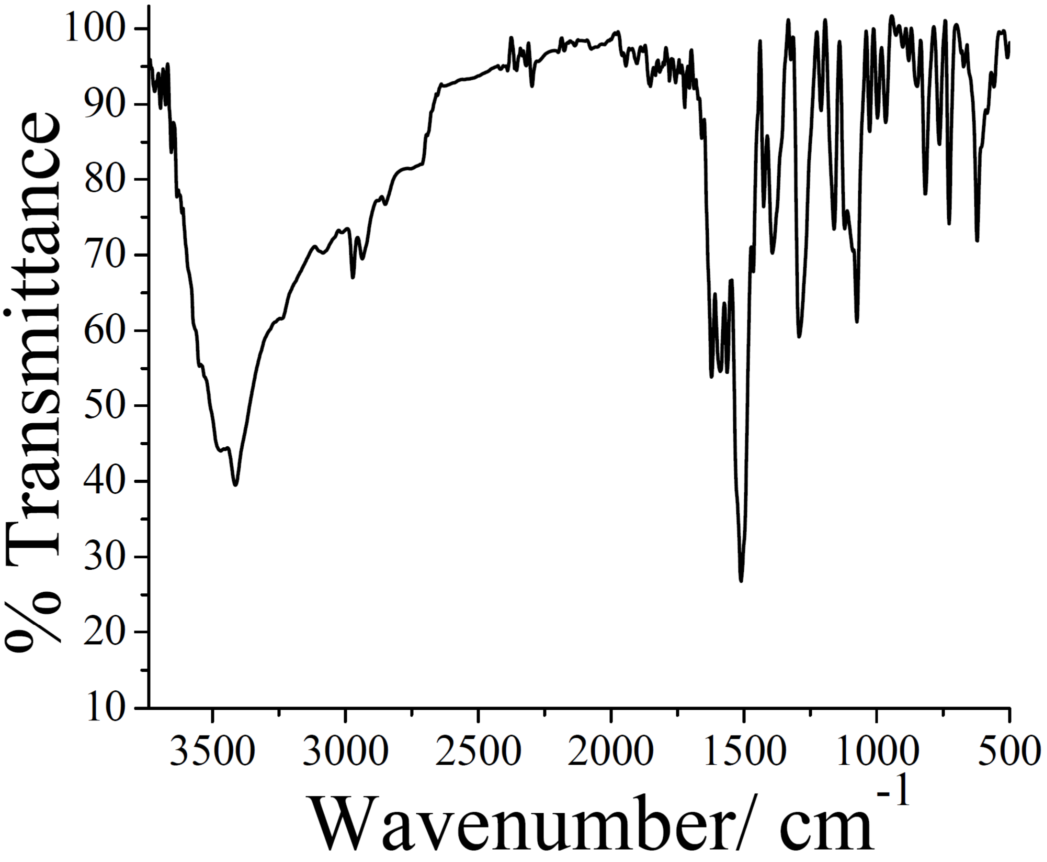

Figure S9. IR spectrum of complex **3**.

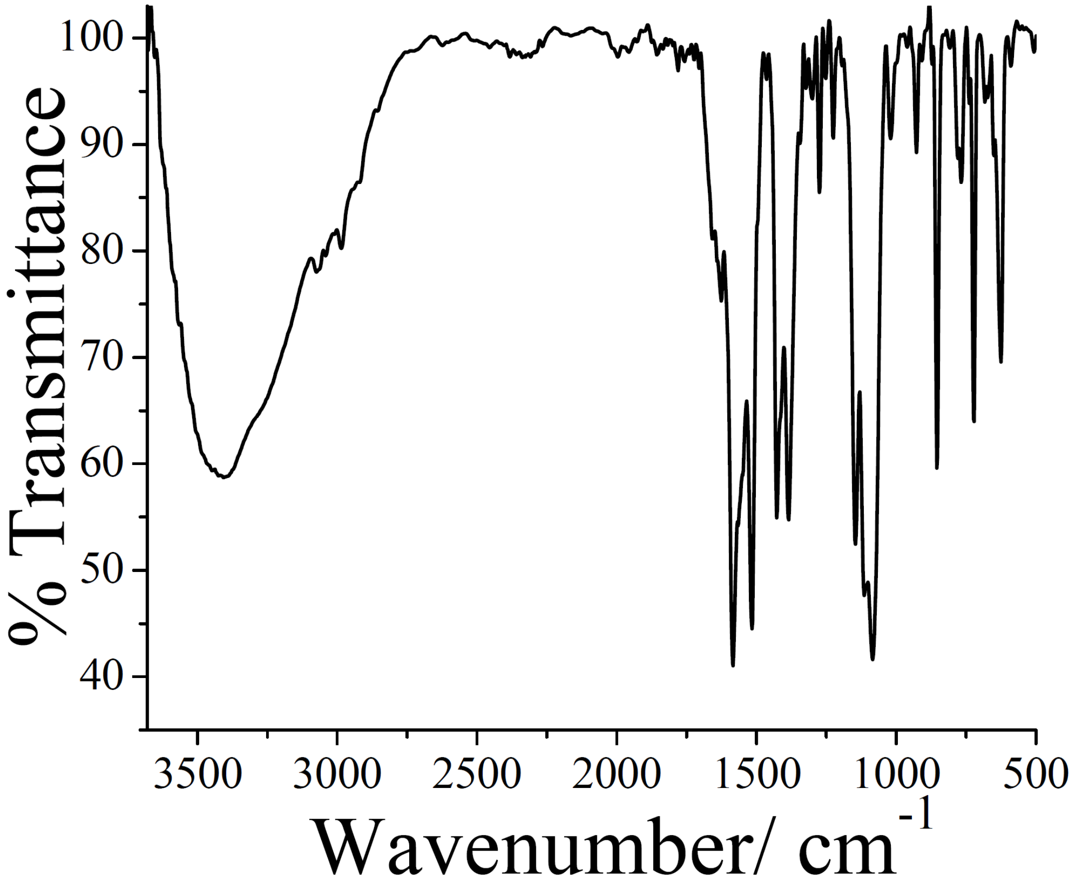

Figure S10. IR spectrum of complex **4**.

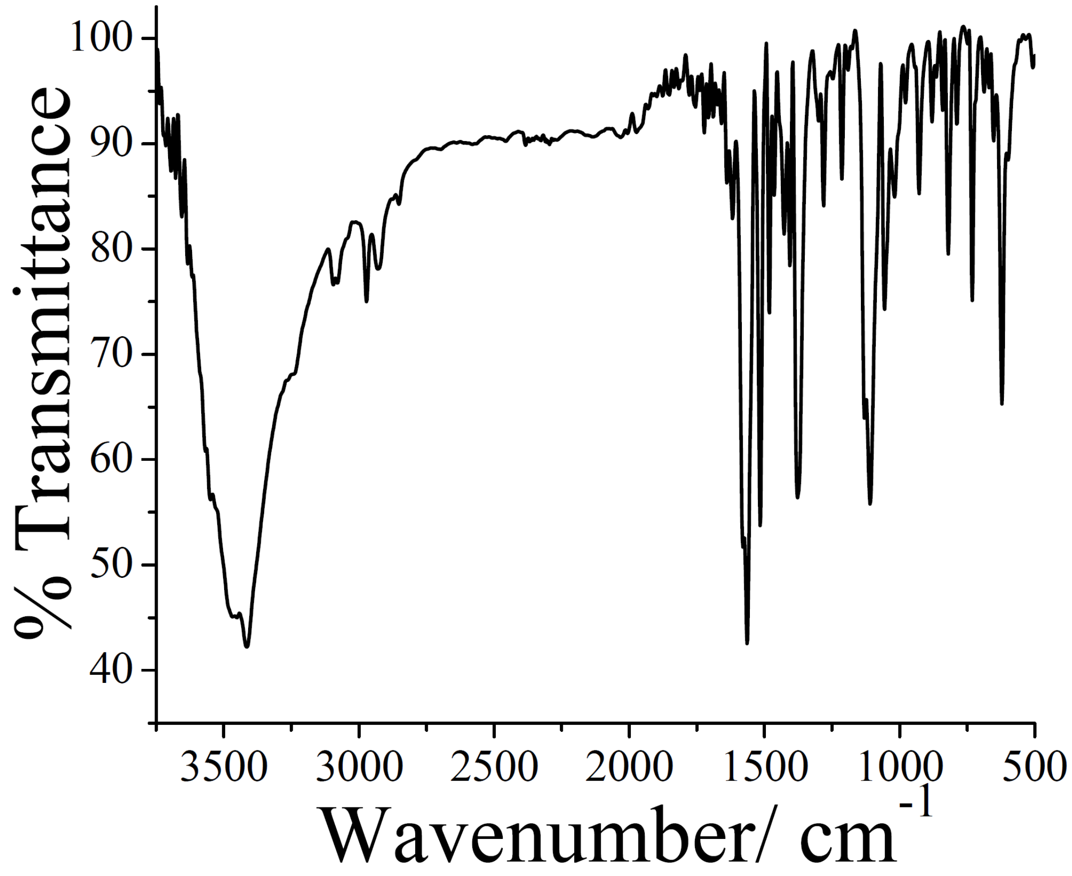

Figure S11. IR spectrum of complex **5**.

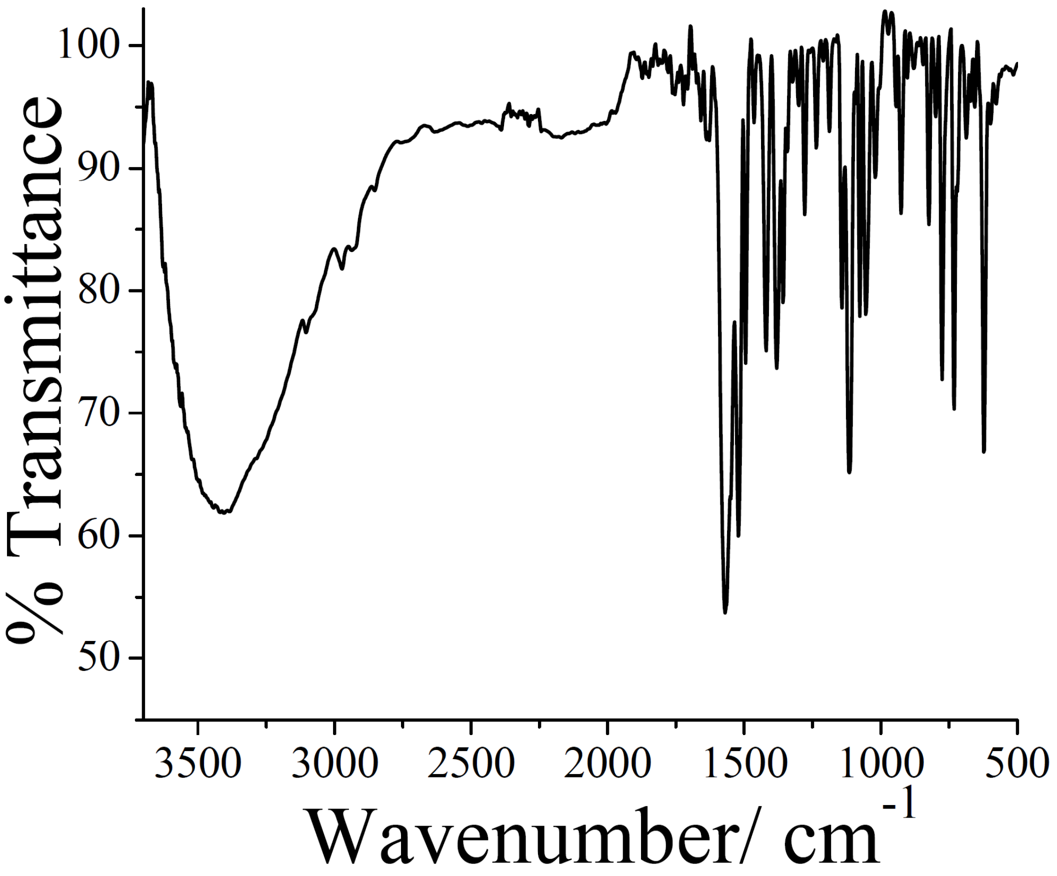

Figure S12. IR spectrum of complex **6**.

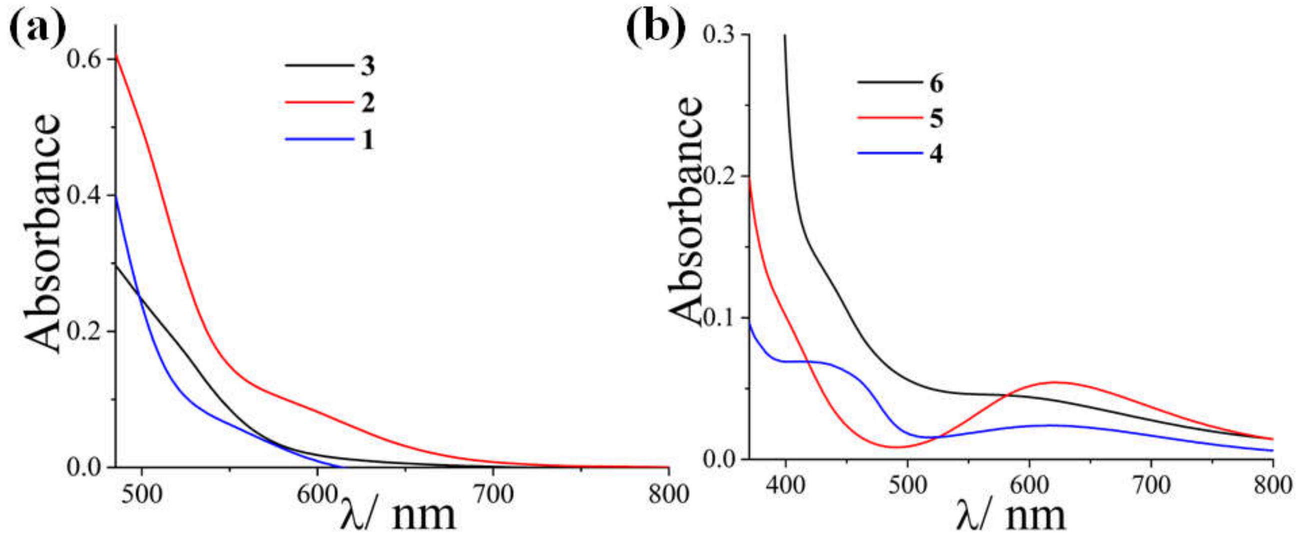

Figure S13. The electronic spectra of the complexes **1-3** (a) and **4-6** (b) (1 mM) in DMF-Tris-HCl buffer (pH 7.2) (1:4 v/v).

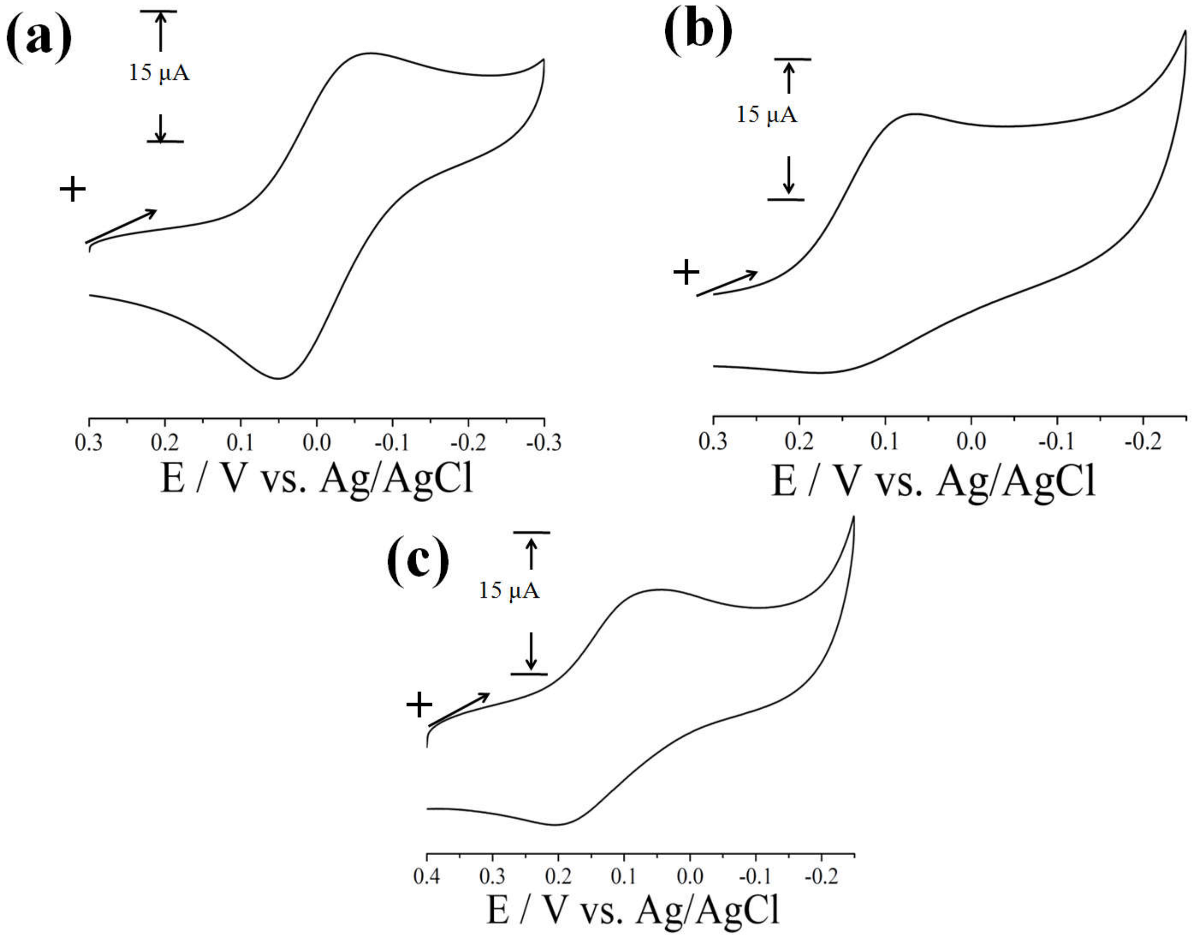

Figure S14. Cyclic voltammograms of complexes **4-6** in DMF using TBAP (0.1 M) as the supporting electrolyte with a scan rate of 50 mV s^{-1}.

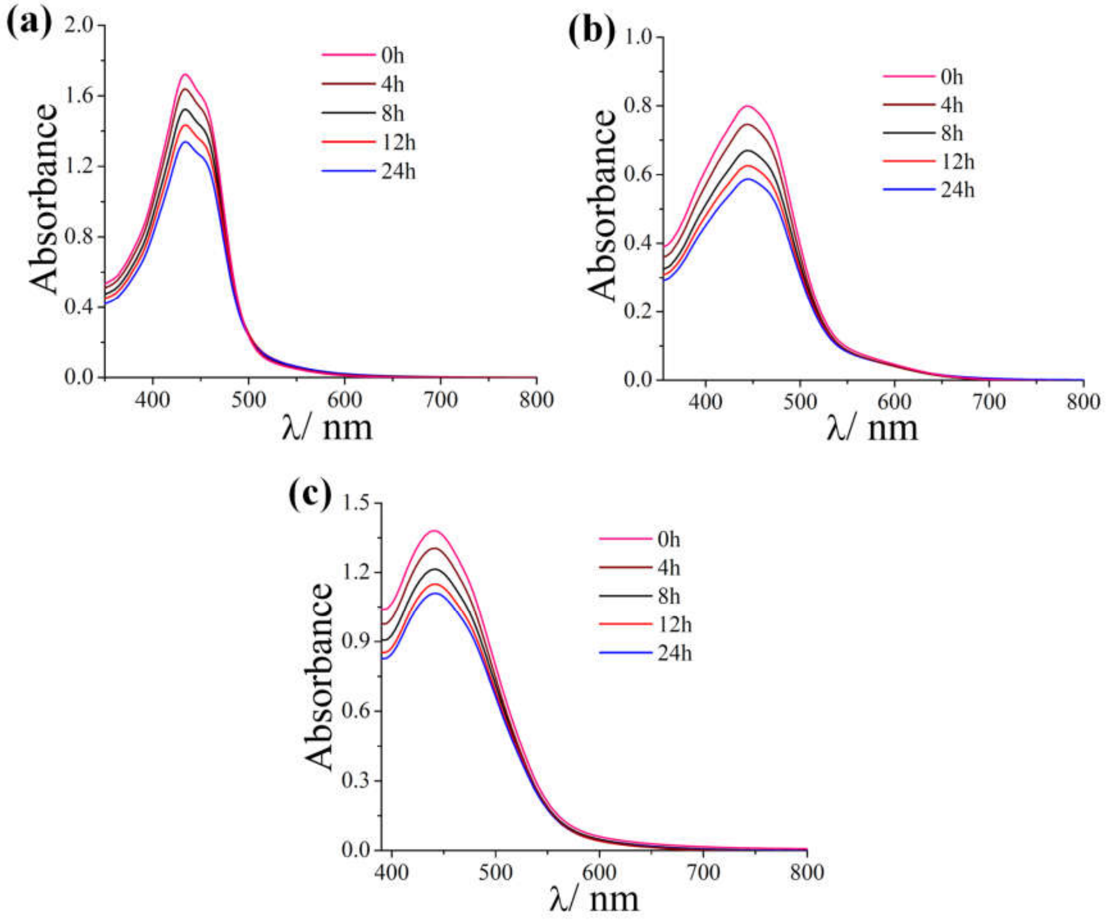

Figure S15. Electronic absorption spectra of the complexes **1-3** (a-c) in DMSO-Tris-HCl buffer (pH 7.2) (1:4 v/v) at different time points showing the solution phase stability of the complexes.

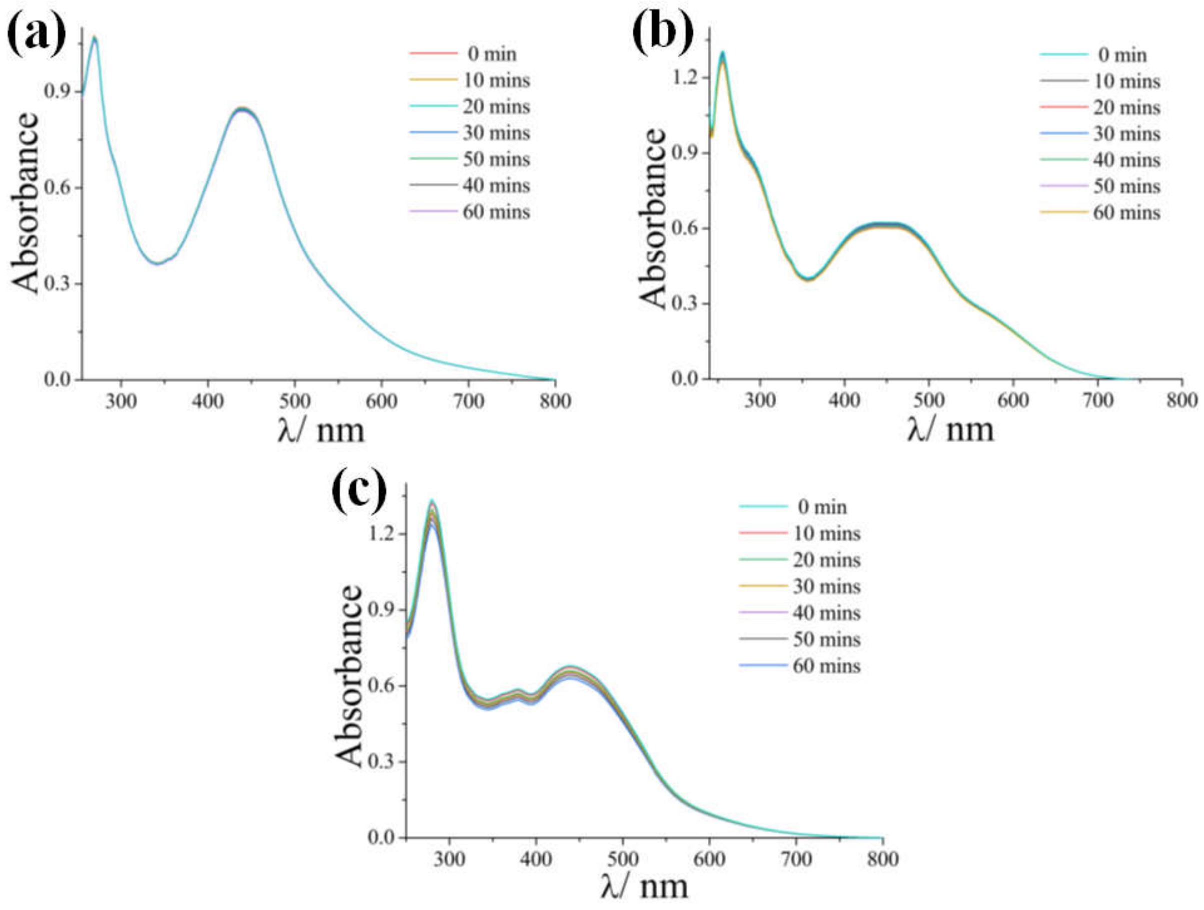

Figure S16. Electronic absorption spectra of the complexes **1** (a), **2** (b) and **3** (c) in DMSO-Tris-HCl buffer (pH 7.2) (1:4 v/v) at different time points of exposure to visible light (400-700 nm).

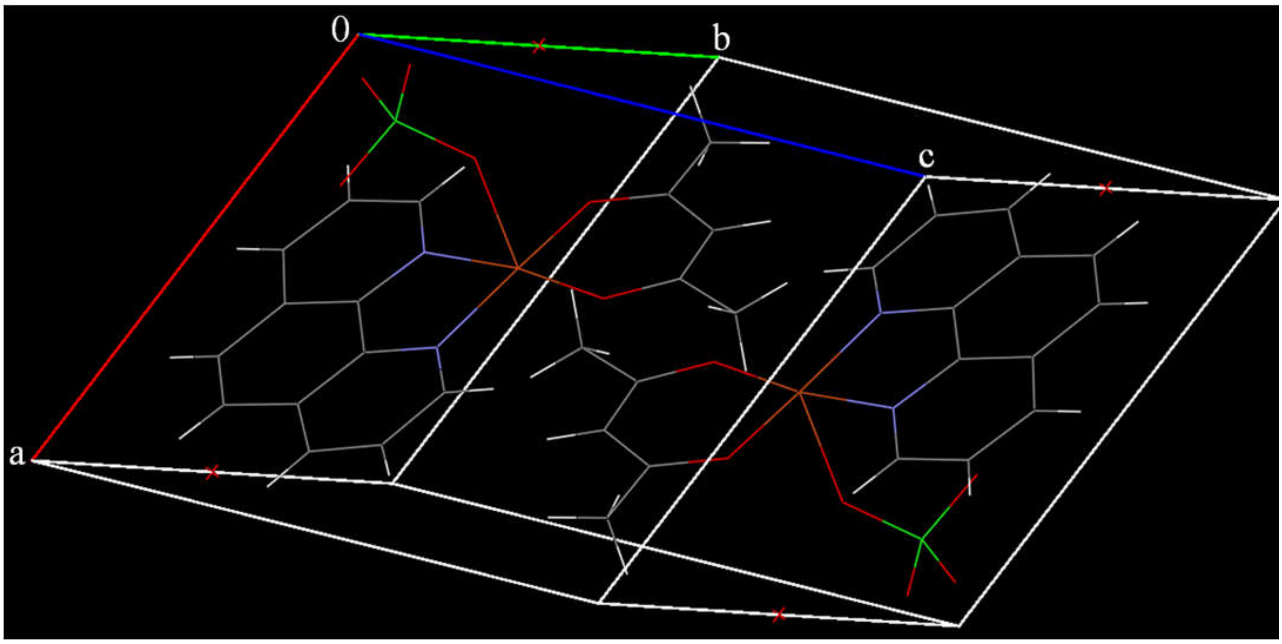

Figure S17. The unit cell packing diagram of [Cu(acac)(phen)(ClO_4)] (**4**) having two molecules in the unit cell.

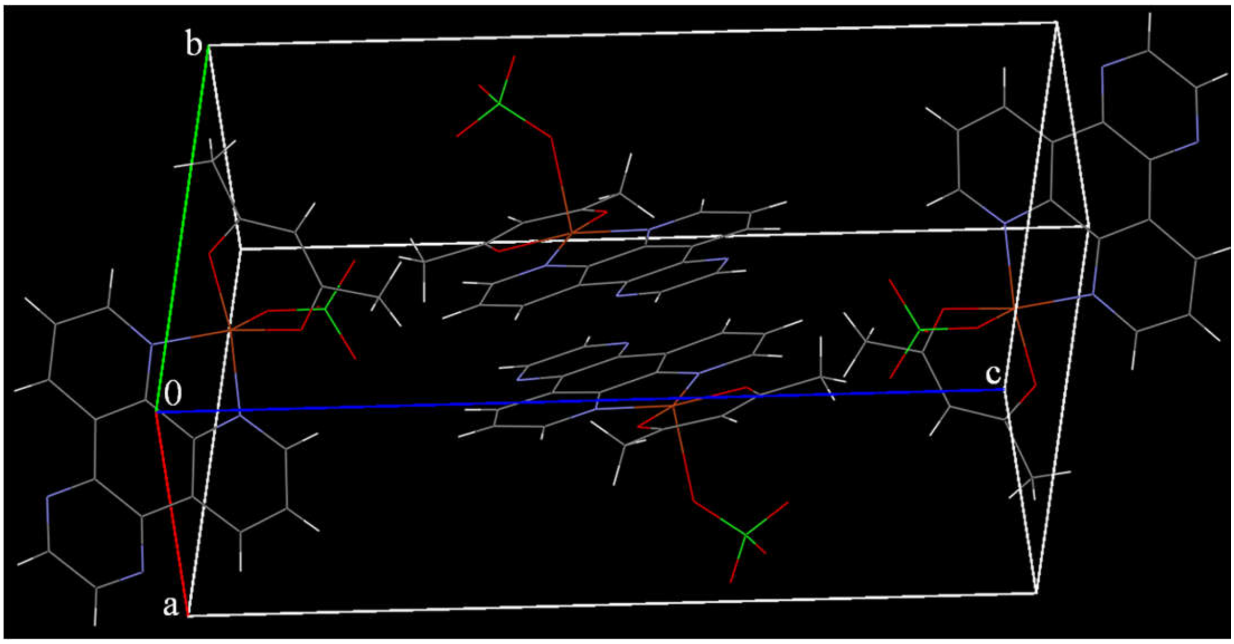

Figure S18. The unit cell packing diagram of [Cu(acac)(dpq)(ClO_4)] (**5**) having four molecules in the unit cell.

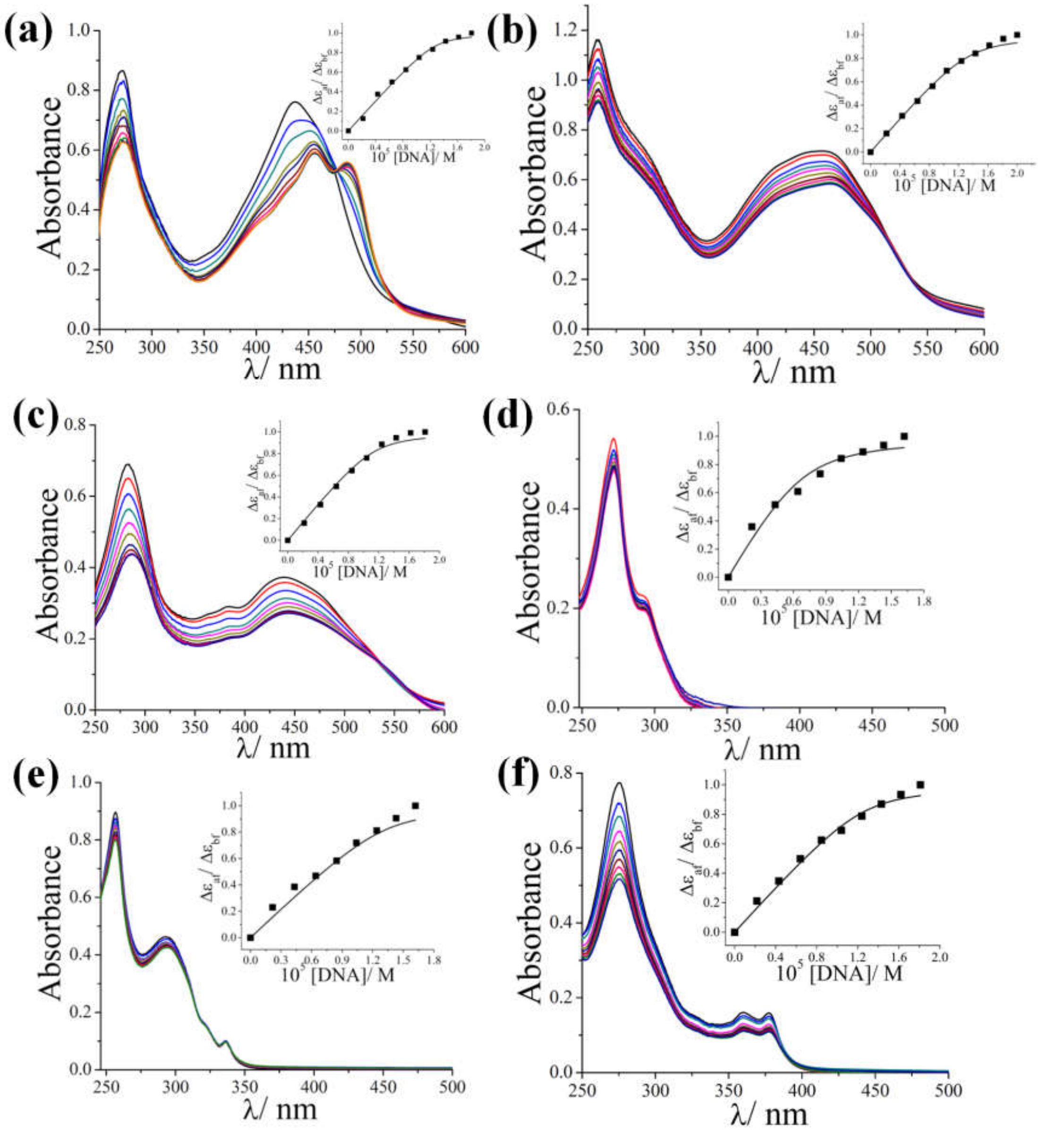

Figure S19. Absorption spectral traces of the complexes **1-6** (**a-f**) in 5 mM Tris-HCl buffer (pH 7.2) on increasing the quantity of calf thymus DNA. The inset shows the least square fits of $\Delta\varepsilon_{af}/\Delta\varepsilon_{bf}$ vs. [DNA] for the complexes using McGhee-von Hippel (MvH) method.

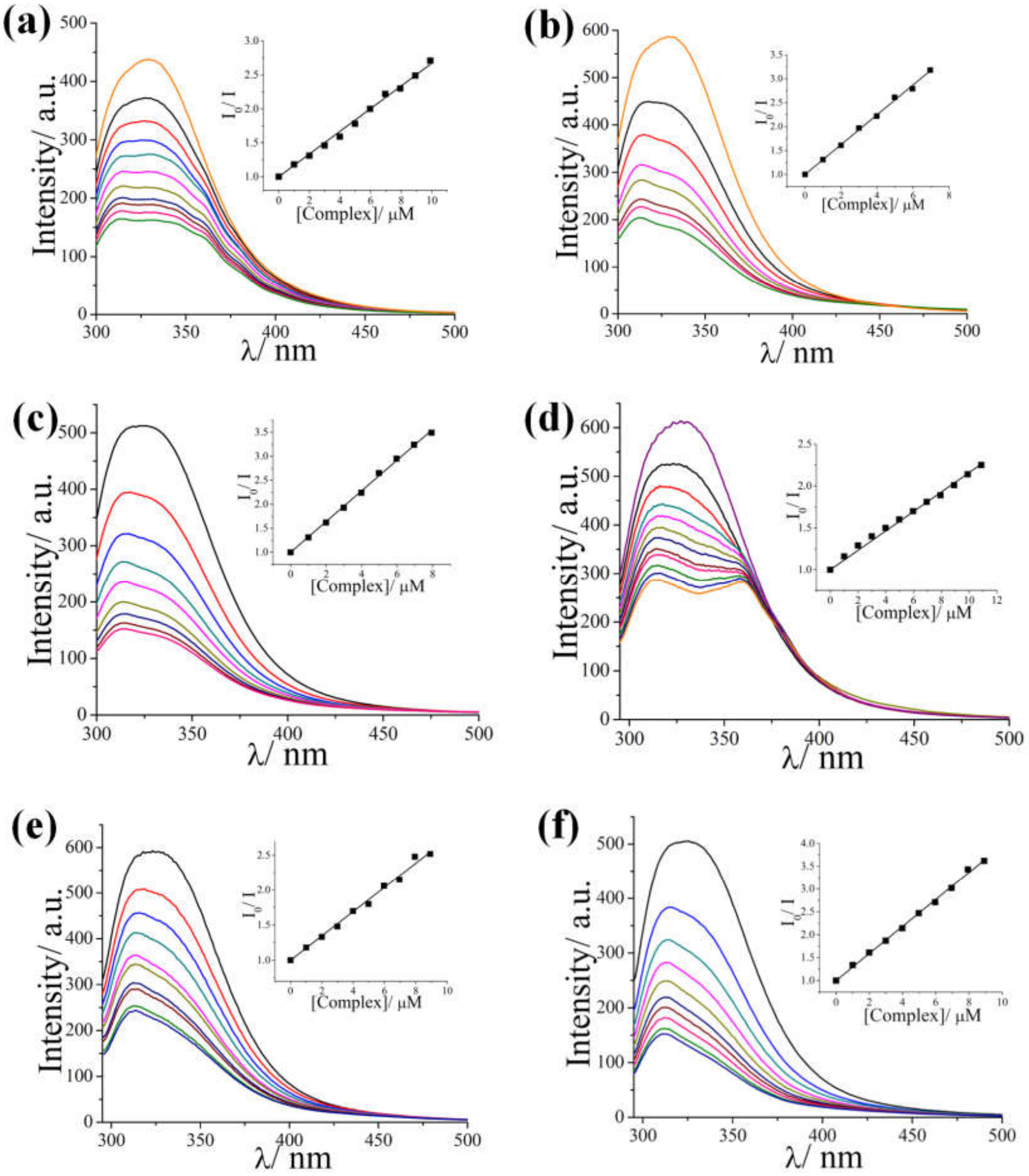

Figure S20. Emission spectral traces of HSA (2 μM) in the presence of complexes **1-6** (**a-f**). The inset shows the plot of (I_0/I) vs. [complex].

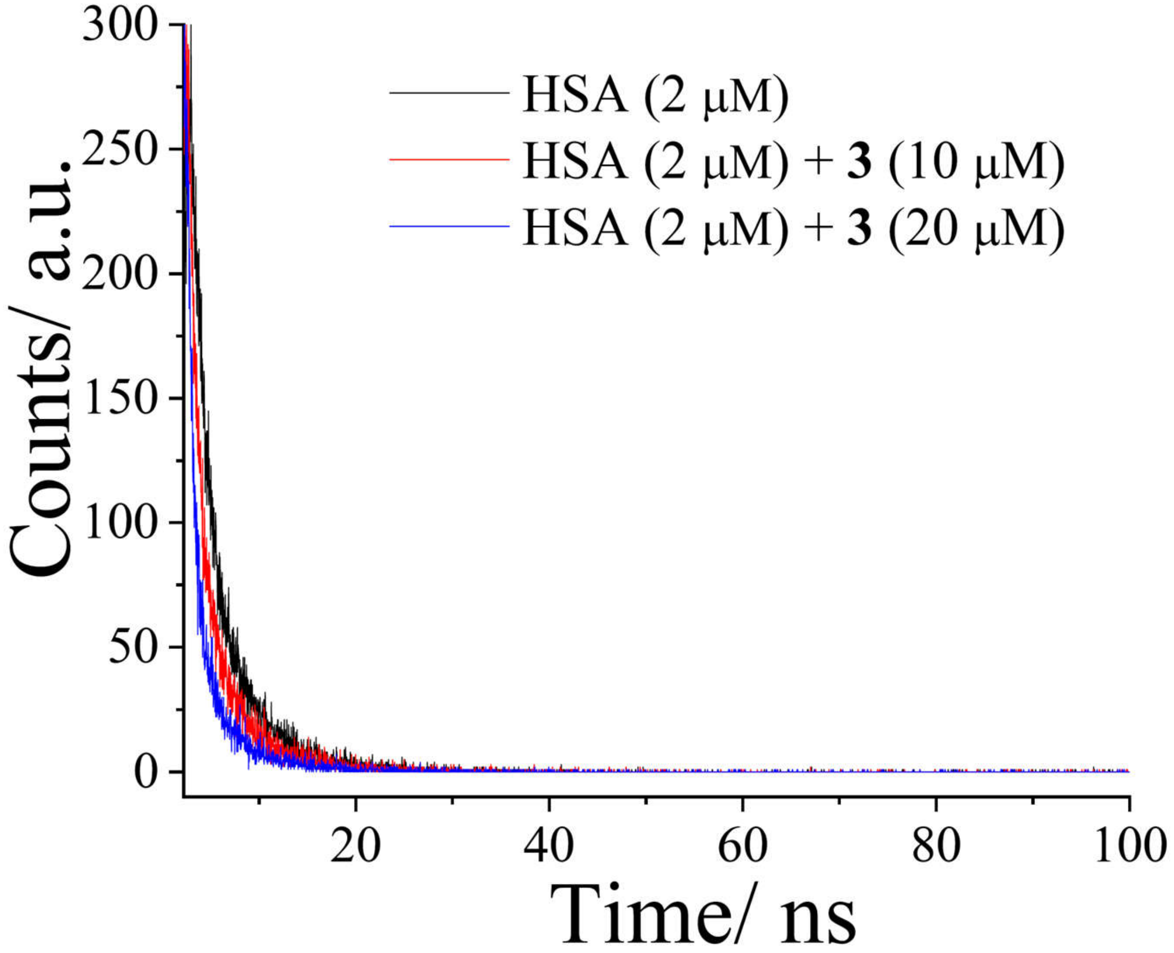

Figure S21. Fluorescence intensity decay profiles of HSA in the absence and presence of 10 and 20 μM of complex **3** in phosphate buffer (pH 7.4).

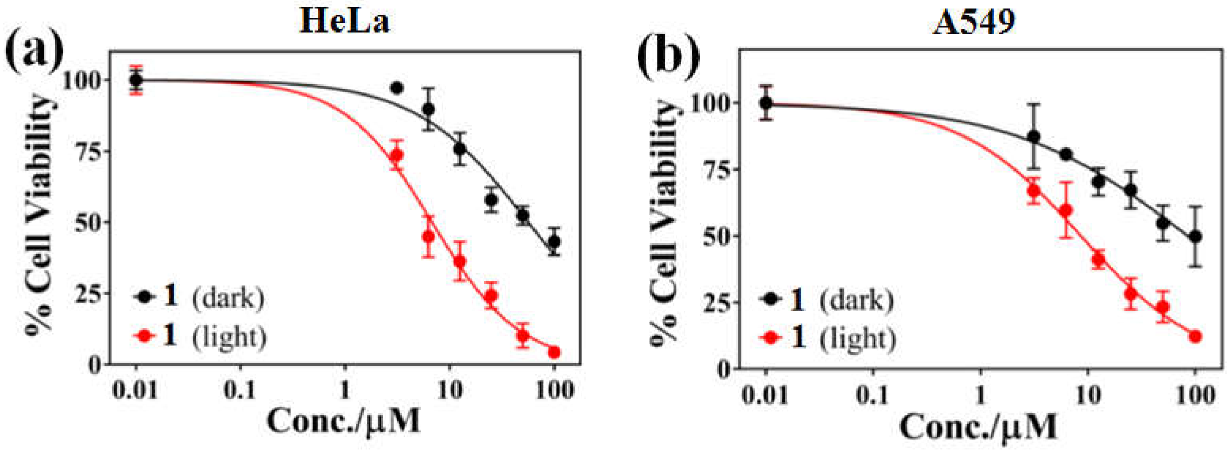

Figure S22. Cell viability plots showing the cytotoxic effect of complex **1** in HeLa (a) and A549 (b) cells in dark (black symbols) and in the presence of visible light (red symbols, 400-700 nm, 10 J cm^{-2}, 1 h).

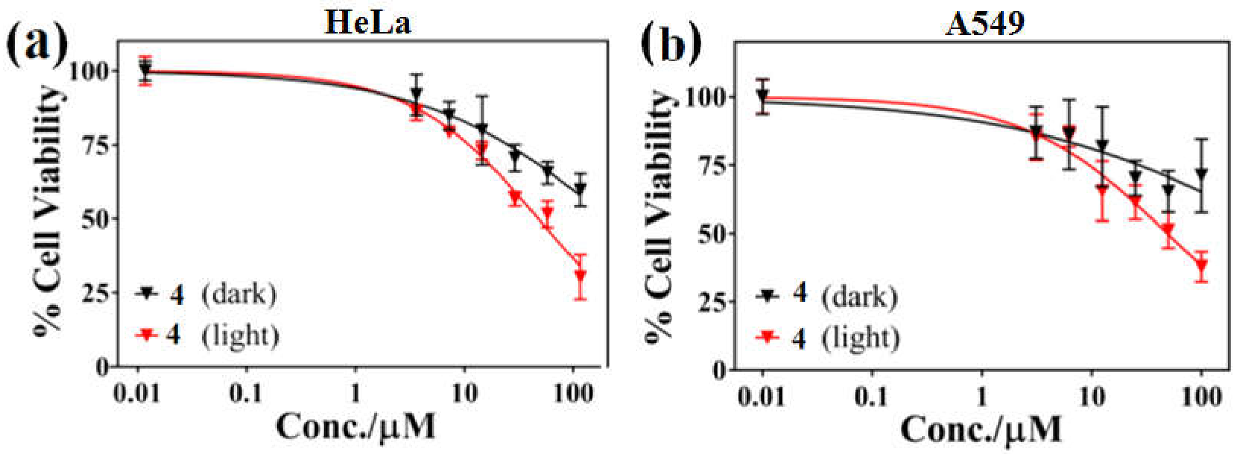

Figure S23. Cell viability plots showing the cytotoxic effect of complex **4** in HeLa (a) and A549 (b) cells in dark (black symbols) and in the presence of visible light (red symbols, 400-700 nm, 10 J cm^{-2}, 1 h).

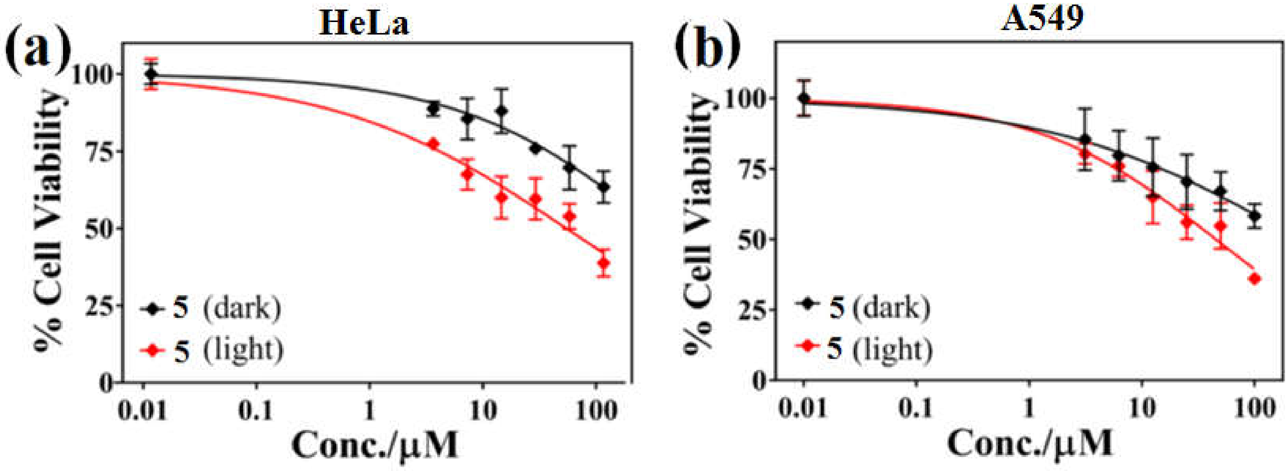

Figure S24. Cell viability plots showing the cytotoxic effect of complex **5** in HeLa (a) and A549 (b) cells in dark (black symbols) and in the presence of visible light (red symbols, 400-700 nm, 10 J cm^{-2}, 1 h).

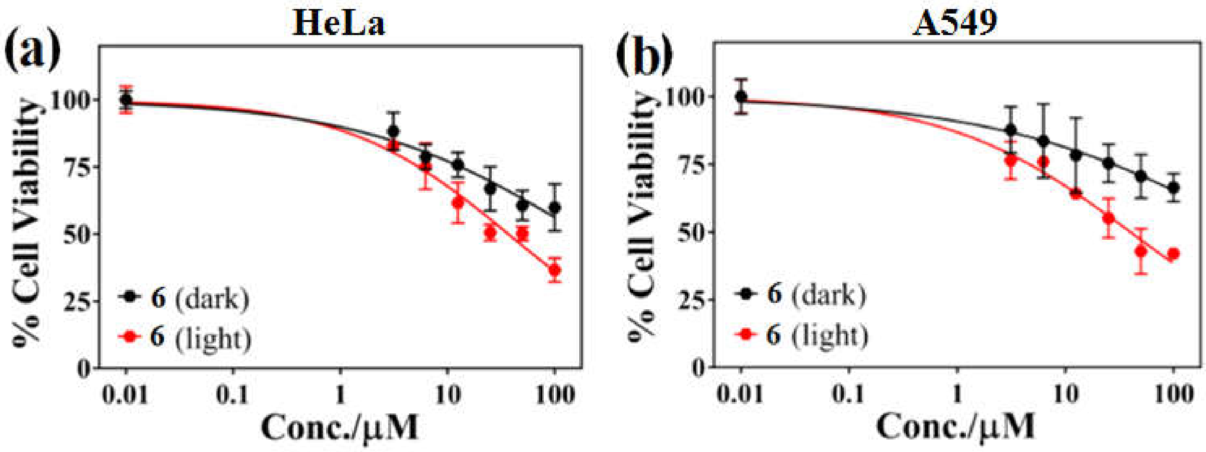

Figure S25. Cell viability plots showing the cytotoxic effect of complex **6** in HeLa (a) and A549 (b) cells in dark (black symbols) and in the presence of visible light (red symbols, 400-700 nm, 10 J cm^{-2}, 1 h).

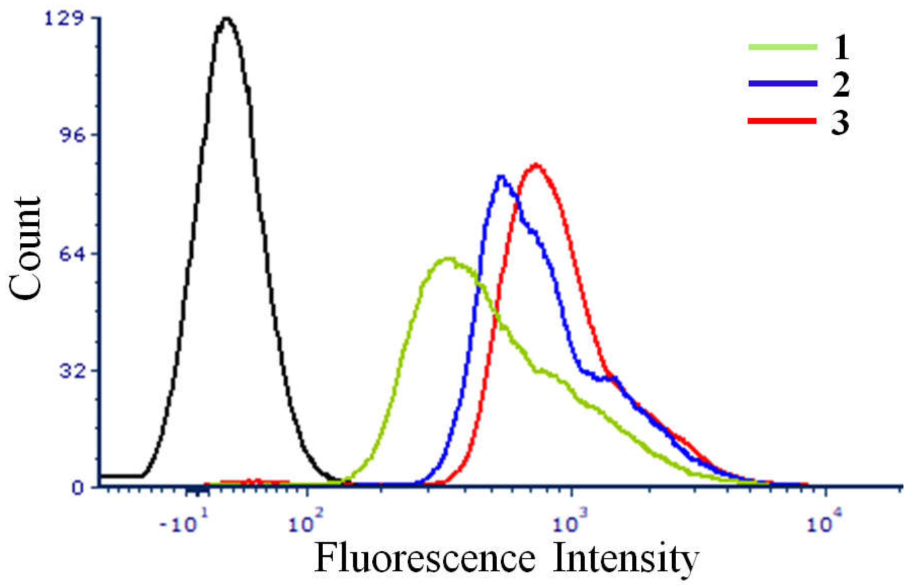

Figure S26. Uptake of the complexes **1**-**3** (10 μM) at 37 °C for 4 h incubation time in HeLa cells determined by flow cytometry. The black line indicates cells' auto fluorescence.

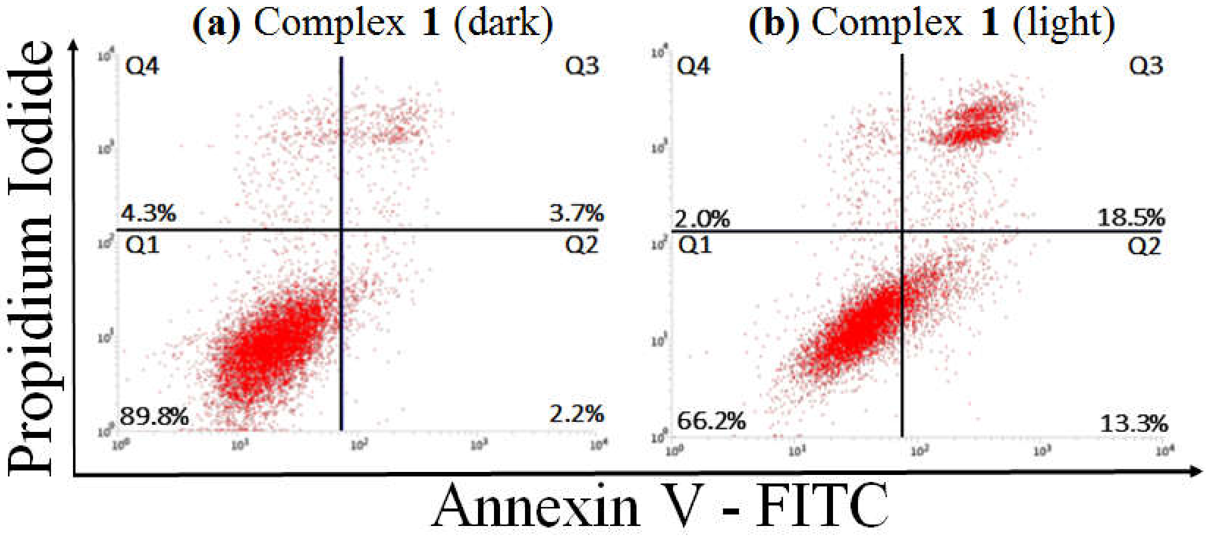

Figure S27. Annexin V-FITC-PI staining of HeLa cells undergoing apoptosis induced by complex **1** (5 μM) in dark and visible light (400-700 nm, 10 J cm^{-2}) analyzed by flow cytometry.

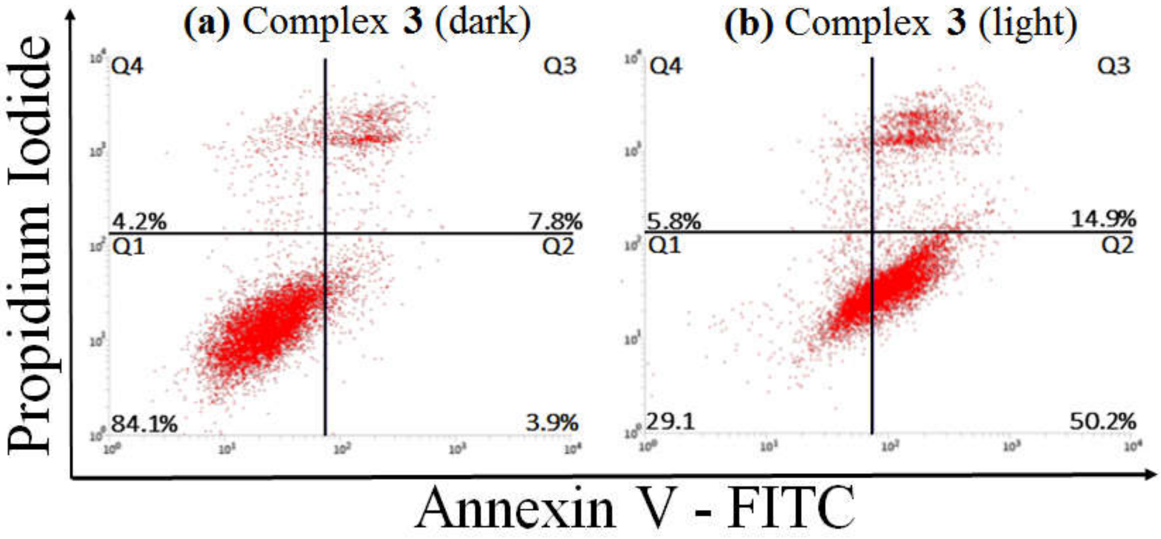

Figure S28. Annexin V-FITC-PI staining of HeLa cells undergoing apoptosis induced by complex **3** (5 μM) in dark and visible light (400-700 nm, 10 J cm^{-2}) analyzed by flow cytometry.
